# High-entropy nanoalloys anchored on entropy-compensating two-dimensional oxides for enhanced nanomagnetism

**DOI:** 10.1126/sciadv.adv8411

**Published:** 2025-11-21

**Authors:** Xiaodi Zhou, Yiqian Du, Huibin Zhang, Bangxin Li, Xuhui Xiong, Xiaowei Lv, Mingyue Yuan, Yihao Liu, Guanyu Chen, Jiacheng Cui, Jian Wang, Jiajun Liu, Hualiang Lv, Renchao Che

**Affiliations:** ^1^ Laboratory of Advanced Materials, Institute of Optoelectronics, Fudan University, Shanghai 200438, P. R. China.; ^2^ Department of Chemistry and Shanghai Key Laboratory of Molecular Catalysis and Innovative Materials, Fudan University, Shanghai 200438, China.

## Abstract

Mitigating detrimental surface effects in nanometals is crucial for advancing applications in quantum magnetic storage and integrated circuits, as size reduction often experiences atomic distortion and vacancy formation, disrupting magnetic domains and electronic transport. Herein, we synthesize nano–high-entropy alloys (HEAs) anchored on two-dimensional high-entropy oxide nanosheets. By leveraging interfacial entropy compensation and the high-entropy effect, the triggered atomic rearrangements alleviate the inhomogeneous stress distribution in magnetic nanoparticles by mitigating unsaturated coordination and surface defects and reinforcing internal distortion, thereby suppressing surface effects. The anchored HEAs demonstrate stable sub–20-nanometer vortex magnetic domains, achieving an 80% enhancement in saturation magnetization and a 135% improvement in permeability, surpassing isolated HEAs and conventional ferromagnetic metals. Moreover, this enhancement enables more than 50% absorption efficiency across the wireless communication spectrum (3.3 to 6.0 gigahertz) with superior thermal stability (300 to 800 kelvins). This study establishes a pathway to enhance nanometal magnetoelectric properties for flexible, high-performance electromagnetic devices.

## Teaser

Sub–20-nm vortex magnetic domains form in nanoalloys via entropy strategies, boosting nanomagnetism and thermal stability.

## INTRODUCTION

Rapid advancements in emerging fields, such as micro/nano-optoelectronics (*1*, *2*), quantum magnetic storage and computing (*3*, *4*), and biomedical imaging and drug release (*5*, *6*), necessitate the development of high-performance nanomaterials. As material dimensions shrink to the nanoscale, the constraint on electron motion and the disruption of atomic configuration imposed by physical boundaries become progressively evident, leading to pronounced surface effects, particularly in nanometals with tens of nanometers (*7*). These surface effects lead to disordered atomic arrangements, lattice distortions, surface defects, and unsaturated coordination, ultimately enhancing the localization and influencing various aspects of electron spin states at surface atoms, including spin-orbit coupling, spin flipping, and spin polarization (*8*, *9*). Subsequently, these alternations considerably degrade the magnetoelectric properties of nanometals by weakening the magnetic exchange interactions, perturbing the intrinsic magnetic anisotropy, inhibiting the formation of stable magnetic domains, and creating discrete energy levels that amplify electron localization and therefore introduce band gaps (*10*–*12*). Furthermore, the random atomic arrangements of nanometals markedly increase the surface energy, which promotes their reactivity with oxygen, making them susceptible to oxidation and leading to poor stability (*13*). Consequently, these size-dependent effects degrade the physical properties of nanomaterials, preventing them from preserving the metallic characteristics typical of bulk materials in practical applications and ultimately causing performance failure. Although mitigating the detrimental surface effects on nanoscale metals and developing high-performance nanomaterials with enhanced magnetic, electrical, and stability properties are crucial, these challenges remain substantial and demand innovative solutions.

High-entropy alloys (HEAs) comprise more than five metal elements randomly distributed in a solid-solution state (*14*, *15*). The coexistence of multiple metal atoms with varying radii within the same crystal structure induces the high-entropy effect. This effect encompasses notable internal lattice distortion, driven by fluctuations in atomic sites and variations in atomic interactions, that reinforces the tolerance to external stress stimuli and enhances mechanical characteristics (*16*, *17*). In addition, the presence of distinct atomic coordination environments and internal lattice distortions suppresses the thermal diffusion and reactions with the environment, resulting in great thermal stability and corrosion resistance (*18*). These effects endow HEAs with unique properties, distinguishing from conventional metals and alloys. When the particle size is reduced to the nanoscale, the internal lattice distortions may become more pronounced, potentially interacting with surface effects and influencing the surface atomic arrangement (*19*–*21*). This could reduce the inherent stress-strain differences between the interior and exterior surfaces in nanoalloys, improving the consistency and continuity of the lattice at the edges and ultimately promoting a more ordered atomic arrangement throughout the nanoalloy, resembling bulk materials but with fewer defects. Therefore, even at scales as diminutive as a few nanometers, HEAs could exhibit minimal hindrance to ordered magnetic domain formation, reduce electronic scattering and localization, and preserve the metallic characteristics with enhanced stability. However, because of the unsaturated coordination environment and high surface energy, existing high-entropy metal nanoparticles continue to display localized lattice distortions and atomic vacancies at the surface, which prevents the full recovery and enhancement of their metallic properties, presenting a notable challenge for their further optimization.

An alternative strategy could be interface compensation, which involves constructing abundant heterointerfaces to neutralize the surface energy of nanometals. This approach could introduce additional interfacial interactions to reduce lattice distortions and defects at nanoparticle edges while alleviating the adverse surface effects on nanoscale properties (*22*, *23*). Moreover, by implementing an entropy-increasing strategy on one or both phases that construct the heterointerface, the enlarged interface entropy can induce an increase in atomic diversity and disturbance of atomic sites (*24*). These modifications may result in more complex and diverse atomic interactions and regulate the electronic structure of the heterointerfaces, thereby strengthening interfacial interactions and further amplifying the compensatory mechanism to mitigate the adverse size-dependent effects. Hence, we propose that high-entropy nanometals anchored onto surfaces with high interface entropy, through the synergistic interplay of interfacial compensation and the alloy’s high-entropy properties, could fully mitigate the detrimental surface effects on nanoalloys, resulting in physical properties that surpass those exhibited by bulk materials.

In this study, we present a strategy to construct entropy-enhanced interfaces around high-entropy magnetic nanoalloys using the in situ growth of uniformly dispersed HEAs anchored on two-dimensional (2D) high-entropy oxide (HEO) nanosheets. The synergistic interaction between interfacial entropy and the intrinsic high-entropy effect eliminates surface defects and promotes atomic rearrangement. These effects generate uniform stress fields throughout nanoalloys and enhance the overall order and continuity of nanometals’ lattice, effectively suppressing the size-dependent effects. Herein, the anchored HEA nanoparticles exhibit enhanced magnetic properties, including stable sub–20-nm vortex magnetic domains and strong magnetic coupling through stray field interactions. Moreover, the strengthened interfacial interactions induced by the entropy increase, coupled with the intrinsic high-entropy state of nanoalloy, provide remarkable thermal and chemical stability. These improvements surpass the performance of isolated HEA nanoparticles and conventional ferromagnetic bulk materials. Thus, the superior magnetic properties of the system with nano-HEAs anchored on HEO nanosheets offer substantial potential for advanced applications, including magnetic refrigeration, quantum magnetic storage, and electromagnetic (EM) functional devices.

## RESULTS

### Synthesis of 2D HEO substrates

**Fig. 1.**
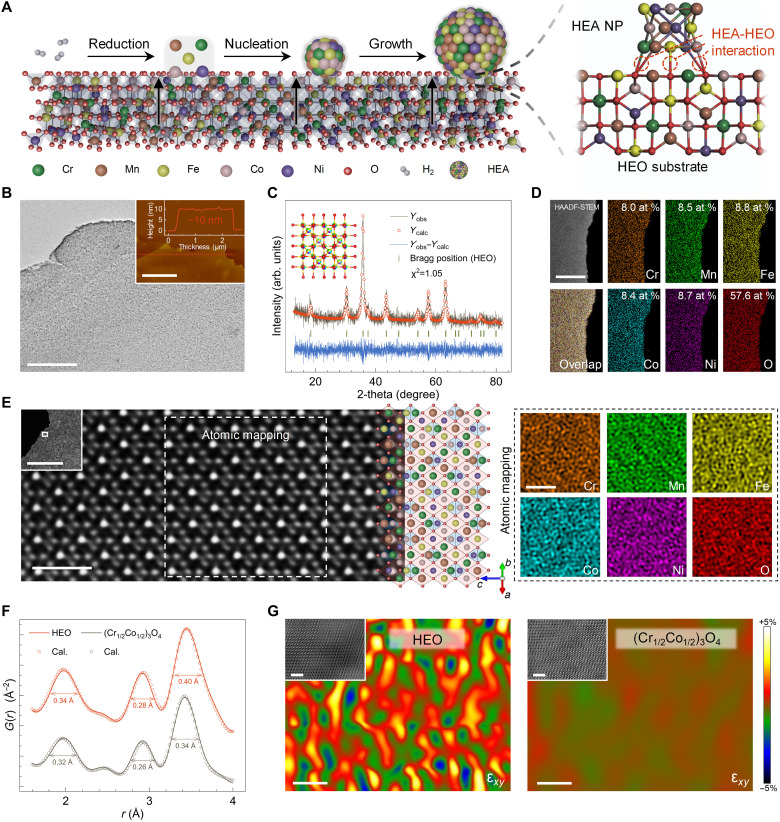
Material characterization of 2D HEO nanosheets. (**A**) Schematic depiction of the in situ growth process of HEA nanoparticles (NPs) on the HEO substrate. (**B**) TEM image. The inserted diagram is the AFM image. (**C**) Rietveld refinement of the XRD pattern. The gray lines, red cycles, green segments, and blue lines correspond to the experimental data, refined pattern, difference, and Bragg position, respectively. (**D**) Elemental mapping of Cr, Mn, Fe, Co, Ni, and O on a single nanosheet. (**E**) The HAADF-STEM image and atomic EDS mapping viewed down the [110] zone axis showing the atomic structure. (**F**) The PDF patterns of HEO and (Cr_1/2_Co_1/2_)_3_O_4_ nanosheets and the FWHM of each diffraction peak are marked. (**G**) Corresponding strain fields ε*_xy_* of HEO and (Cr_1/2_Co_1/2_)_3_O_4_ nanosheets via the GPA method, with a color scale from −5 to +5%. Scale bars: (B) 200 nm and (inset) 1 μm, (D) 200 nm, (E) 1 nm and (inset) 200 nm, and (G) 2 nm.

This study introduces an efficient, controllable method for fabricating HEA nanoparticles on ultrathin 2D HEO nanosheets, leveraging an entropy-compensated interfacial interaction that, in synergy with the metallic high-entropy properties, mitigates the detrimental surface effects on nanometals. As shown in Fig. 1A and fig. S1, the nano-HEAs anchored on HEO nanosheets are fabricated via two key steps: (i) synthesis of 2D oxide nanosheets using a self-template method and (ii) in situ growth of HEA nanoparticles anchored on HEO substrates via a thermal reduction treatment. Notably, this method is based on simple solid-state sintering and enables the batch production of several grams (fig. S2), thereby demonstrating its scalability and providing feasibility for future practical applications.

For fabricating the anchored HEAs, the 2D HEO nanosheet precursor is initially prepared. The ultrathin nanosheet morphology of the precursor, with the thickness of merely 10 nm, is confirmed using field-emission scanning electron microscopy (SEM), transmission electron microscopy (TEM), and atomic force microscopy (AFM) (Fig. 1B and fig. S3). The formation of this morphology can be attributed to the assembly of 2D polyvinylpyrrolidone (PVP) micelles decorated with metal ions via freeze-drying treatment, followed by the self-decomposition of the layered PVP templates during the subsequent foaming procedure. The crystal information is examined via the x-ray diffraction (XRD) and electron diffraction patterns. As illustrated in Fig. 1C and fig. S4, the 2D nanosheet exhibits a single phase of a typical cubic spinel oxide without any impurities. In addition, the synthesized nanosheet displays a polycrystalline state, assembled from tiny nanocrystals, which clarifies the formation mechanism of its nanoscale thickness. The uniform distribution of the five metal elements, Cr, Mn, Fe, Co, and Ni, at equivalent molar ratios and oxygen across the lamellar nanostructure is determined via high-angle annular dark-field scanning transmission electron microscopy (HAADF-STEM) and the corresponding energy-dispersive x-ray spectroscopy (EDS) elemental mapping (Fig. 1D). Specifically, the atomic ratios of total metal elements (42.3%) and oxygen (57.6%) closely approximate the theoretical value of three-fourths in an ideal spinel oxide phase. As revealed using x-ray photoelectron spectroscopy (XPS), all five metal elements exhibit divalent and trivalent states (fig. S5) (*25*). Therefore, it is proposed that the crystal structure of 2D nanosheets corresponds to a cubic spinel oxide phase, with each of the five metal elements holding one-fifth occupancy at both tetrahedral and octahedral lattice sites.

The atomic-resolution HAADF-STEM image, coupled with the corresponding EDS mapping, is used to elucidate the crystalline information at the atomic scale. Figure 1E presents the atomic arrangement of the precursors viewed along the [110] zone axis. This configuration is consistent with a spinel lattice belonging to the group space ( $\mathrm{Fd}\bar{3}m$ ). The accompanying elemental mapping demonstrates the uniform distribution of cations and anions on the atomic scale without any discernible separations, indicating the homogeneous distribution of various metal ions in the tetrahedral and octahedral sites (*26*). Overall, the 2D high-entropy spinel oxide precursor is well prepared.

Furthermore, we systematically examine the effects of high-entropy engineering on the spinel oxide lattice and stress-strain distribution. For comparison, (Cr_1/2_Co_1/2_)_3_O_4_ nanosheets are synthesized using a similar method, incorporating only two metal elements, Cr and Co, instead of five (figs. S6 and S7). These nanosheets exhibit an identical crystalline phase to the HEO precursors. Interatomic distances between neighboring atom pairs are assessed using pair distribution function (PDF) fitting analysis (*27*, *28*). Owing to the complexity of the cubic spinel oxide structure, three diffraction peaks at 1.97, 2.91, and 3.43 Å are selected to evaluate lattice variations (fig. S8). As shown in Fig. 1F, the diffraction positions in the HEO and (Cr_1/2_Co_1/2_)_3_O_4_ samples are nearly identical, indicating the high stability and tolerance of the spinel oxide framework under high-entropy engineering. Nevertheless, the HEO nanosheets exhibit larger full width at half maximum (FWHM) values of 0.34, 0.28, and 0.40 Å than 0.32, 0.26, and 0.34 Å for the (Cr_1/2_Co_1/2_)_3_O_4_ sample. This phenomenon could be ascribed to the coexistence of multiple metal atoms with various radii in the high-entropy system, leading to substantial fluctuations in interatomic distances and larger FWHM values (*29*). Such variations typically induce lattice distortions and intense stress distributions.

To investigate stress fields within the crystal lattice intuitively, we use the geometric phase analysis (GPA) of differential phase contrast TEM. As depicted in Fig. 1G, the HEO nanosheets display more pronounced stress distribution and lattice distortions than (Cr_1/2_Co_1/2_)_3_O_4_, attributing to variations in metal ion radii and perturbations in atomic positions. In summary, the 2D HEO nanosheets maintain a stable crystal framework with enhanced lattice distortions, facilitating the homogeneous precipitation of secondary phases on the nanosheet substrate and alleviating the lattice mismatch that occurs during the subsequent formation of heterointerfaces.

### Entropy-increased interface assisting the growth of high-entropy nanoalloys

Following the preparation of ultrathin HEO nanosheets, the in situ recrystallization of secondary substance is conducted to construct entropy-increased heterointerfaces. In detail, during the high-temperature reduction procedure, metal atoms, initially reduced to zero valences, detach from the bulk HEO nanosheet and spontaneously assemble into HEA nanoparticles on the nanosheet surface. With subsequent grain growth, magnetic HEA nanoparticles are uniformly anchored on the 2D HEO nanosheets via strong interfacial interactions.

**Fig. 2.**
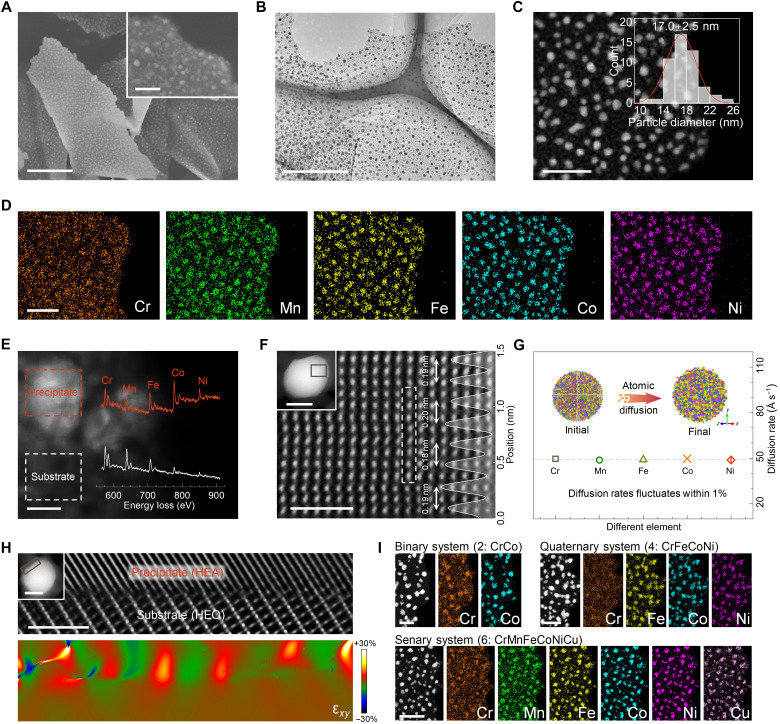
Microscopic characterization of anchored HEA nanoparticles. (**A**) SEM images. (**B**) TEM image. (**C** and **D**) HAADF-STEM image and corresponding EDS mapping. The inset diagram shows the size distribution of precipitated nanoparticles. (**E**) Electron energy loss spectroscopy mapping image with nanoscale resolution. The inset electron energy loss spectroscopy curves are acquired from marked areas. The red color represents the precipitated phase, while the white color represents the bulk substrate. (**F**) HAADF image of precipitated nanoparticles viewed along the [100] direction. The top inset indicates the selected area. The intensity profiles in the white rectangle are exhibited on the right. (**G**) Diffusion rates of Cr, Mn, Fe, Co, and Ni atoms within precipitated nanoparticles during the nucleation procedure, obtained from the molecular dynamic simulation. (**H**) The top shows the HAADF-STEM image with the atomic resolution of the heterointerface between the precipitate and the bulk phase. The selected area is marked in the inset image. Below shows the corresponding stress field ε*_xy_* via the GPA method, with a color scale from −30 to +30%. (**I**) HAADF images and STEM elemental maps of the alloy-on-oxide system with various metal elements: binary (2: CrCo), quaternary (4: CrFeCoNi), and senary (6: CrMnFeCoNiCu). Scale bars: (A) 500 nm and (inset) 100 nm, (B) 200 nm, (C and D) 100 nm, (E) 10 nm, (F) 1 nm and (inset) 10 nm, (H) 2 nm and (inset) 10 nm, and (I) 100 nm.

The morphology of the as-prepared composite is characterized using SEM and TEM images. In Fig. 2 (A and B), the hierarchical architecture of homogeneous nanoparticle growth on the ultrathin nanosheets is demonstrated. As shown in the HAADF-STEM image (Fig. 2C), the precipitated nanoparticles present a uniform size distribution of 17.0 ± 2.5 nm, which could be attributed to the polycrystalline nature of the nanosheet substrate that facilitates the growth of the secondary phase at multiple crystalline planes and simultaneously inhibits the self-agglomeration of the precipitated nanoparticles. The chemical compositions of the bulk substrate and secondary phase are analyzed via EDS elemental mapping. In Fig. 2D and fig. S9, all five metal elements, Cr, Mn, Fe, Co, and Ni, are observed in both the nanosheet substrate and precipitated nanoparticles. In particular, Cr and Mn exhibit a greater propensity to disperse across the substrate, whereas Fe, Co, and Ni tend to concentrate within the recrystallized nanoparticles (fig. S10 and table S1).

The crystalline structure and interatomic distance are investigated in fig. S11. First, both the high-resolution selected area electron diffraction pattern and the Rietveld refinement of the XRD pattern reveal the coexistence of two phases within the synthesized composites: One closes to the typical spinel oxide structure of Fe_3_O_4_ (JSC card no. 19-0629), and another matches the crystal structure of metallic Co (JSC card no. 15-0806). This phase identification is further confirmed with PDF analysis, where an additional diffraction peak at 2.42 Å, belonging to metal atom pairs in the alloys (*30*), is observed after the precipitation of the secondary phase. In addition, the chemical valence is examined using XPS (fig. S12). Except for ionic states observed in the HEO nanosheet precursor, all five metal elements, Cr, Mn, Fe, Co, and Ni, display signals corresponding to their metallic states. On the basis of the above analysis, the initial conjecture of this system is that both oxide substrates and precipitated nanoparticles exhibit the high-entropy solid-solution state.

To further validate this hypothesis, an in-depth characterization concentrated on a single recrystallized nanoparticle is performed. High-resolution EDS mapping reveals that elements of Cr, Mn, Fe, Co and Ni are evenly distributed within a precipitated nanoparticle without any elemental separation or aggregation (fig. S13). Electron energy loss spectroscopy is then performed separately for the nanoparticle and bulk substrate. The distinctive signals corresponding to all metal elements are detected in both regions (Fig. 2E). Regarding the intensity of the characteristic peaks, the proportions of Fe, Co, and Ni in the precipitated nanoparticles are higher than those of Cr and Mn. Conversely, the opposite trend is observed in the bulk oxide nanosheet. In fig. S14, compared with the oxide substrate, all peaks in the spectrum of precipitated nanoparticles shift toward lower energies, suggesting the presence of metallic valence for Cr, Mn, Fe, Co, and Ni elements (*31*).

The atomic arrangements of the precipitation phase and bulk nanosheet are examined via high-resolution HAADF-STEM images. The bulk oxide retains the typical spinel oxide crystalline structure, revealing the crystal state of the HEO phase (fig. S15). For the precipitated nanoparticles, combined with quantitative STEM simulation, all metal atoms occupy lattice sites within the face-centered cubic crystal structure instead of the interstitial spaces, demonstrating the formation of the HEA phase rather than intermetallic substance (Fig. 2F and fig. S16) (*32*). Moreover, fluctuations in the lattice sites and the interatomic distance between adjacent atoms are detected, signifying notable internal lattice distortions within the HEA nanoparticles (*33*). Molecular dynamic simulation further confirms the formation of high-entropy nanoalloys (Fig. 2G and fig. S17). The atomic diffusion rates of Cr, Mn, Fe, Co, and Ni are almost identical, leading to the uniform distribution of multiple metal elements throughout the precipitated nanoparticles and thereby avoiding phase separation.

Furthermore, the stress distribution within the synthesized system is characterized. In fig. S18, a more pronounced stress field is observed in the HEA precipitates in comparison to the HEO substrates, indicating a greater degree of lattice distortions within the nanoalloys. Furthermore, owing to the high lattice similarity between the synthesized oxide substrate and the precipitated nanoalloy, along with the increased tolerance to lattice mismatch during the formation of heterogeneous interfaces caused by atomic position fluctuations in the high-entropy state, a substantial number of entropy-increased heterointerfaces are constructed between the HEA nanoparticles and the HEO nanosheet (fig. S19). As the interfacial regions transition into high-entropy states, the augmented compositional complexity induces local fluctuations in atomic potentials and interatomic distances, expanding the ensemble of energetically degenerate states accessible for atomic rearrangement and consequently facilitating localized structural relaxation while mitigating the driving force for stress accumulation. This evolution alleviates stress disparities at the edges, fostering both thermodynamic and mechanical stabilities (Fig. 2H). For these precipitated nanoalloys, the high-entropy state amplifies the internal stress field, while the interfacial entropy compensation effectively reduces stress-strain in the edge regions. The synergistic interplay of these two factors offers a more homogeneous stress distribution across the HEA nanoparticles and improves the overall lattice ordering (fig. S20). In contrast, isolated HEA nanoparticles exhibit substantial lattice distortions and defects on the surface, causing abrupt stress variations near the edges (figs. S21 to S24). For anchored CoCr nanoalloys, because of their non–high-entropy state and insufficient interfacial interactions, twin structures are formed within nanoalloys, resulting in a nonuniform stress distribution (figs. S25 to S27). Only when both the high-entropy treatment and interfacial entropy compensation strategy are simultaneously implemented can the detrimental surface effects be effectively suppressed. Overall, a system in which HEA nanoparticles are firmly integrated onto HEO nanosheets through enhanced interfacial interactions at entropy-increased heterogeneous interfaces is successfully prepared.

Next, the tunability and generalizability of this system with nanoalloys anchored on oxide nanosheets are explored. First, the size regulation of the precipitated HEA nanoparticles can be achieved by controlling their recrystallization process using various reducing temperatures. In detail, the particle diameter gradually increases from the nanoscale to the submicrometer range (10.2 ± 2.7 nm for 400°C, 29.6 ± 4.2 nm for 600°C, 54.6 ± 5.6 nm for 700°C, and 106.4 ± 10.4 nm for 800°C), while XRD patterns still exhibit the coexistence of the spinel oxide and alloy substances without any phase separation or impurity (figs. S28 to S30). This phenomenon is further explained via molecular dynamic simulation. Within the target temperature range, the tiny differences in diffusion rates among various metal atoms are insufficient to induce elemental separation or partial aggregation in the precipitated nanoparticles, thus resulting in a homogeneous HEA phase (fig. S31) (*34*). Second, the in situ growth of nanoalloys on the corresponding oxide nanosheets to construct heterointerfaces can be implemented iteratively, regardless of the elemental composition. By reducing or adding metal salt types, binary (CrCo) (fig. S25), quaternary (CrFeCoNi) (figs. S32 to S34), and senary (CrMnFeCoNiCu) (figs. S35 to S37) systems with similar nanostructures have been well fabricated, where all metal elements uniformly disperse across oxide nanosheets and embodied nanoparticles (Fig. 2I). A prerequisite for this approach is that at least one relatively less reactive element, such as Cr and Mn, should be selected to prevent the collapse of the lamellar structure of the oxide substrate during the thermal reduction treatment (fig. S38).

### Analysis of magnetic properties in anchored high-entropy nanoalloys

**Fig. 3.**
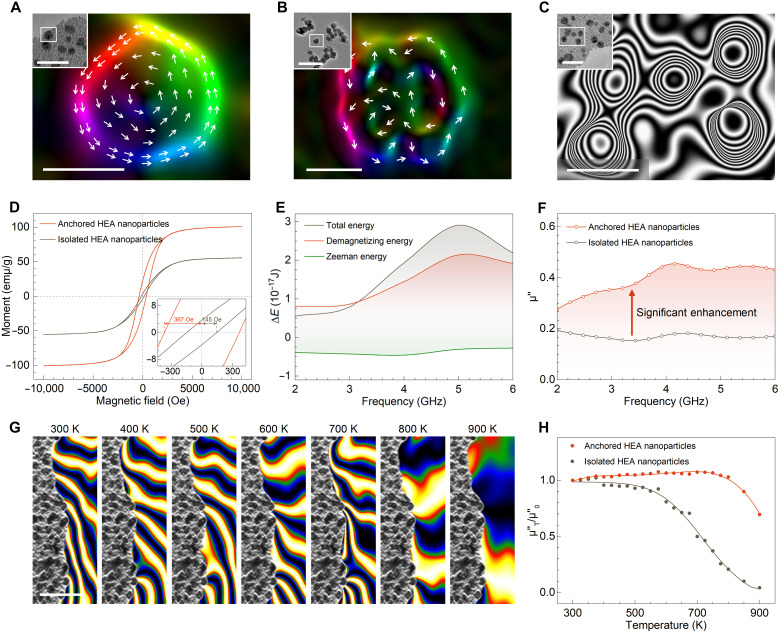
Magnetic properties of anchored HEA nanoparticles. (**A** and **B**) Off-axis electron holography images of magnetization distribution for (A) anchored HEA nanoparticles and (B) isolated HEA nanoparticles. (**C**) Off-axis electron holography images of outer magnetic lines flux among anchored HEA nanoparticles. (**D**) Magnetization-coercivity (*M*-*H*) curves for anchored and isolated HEAs. The enlarged inset shows corresponding coercivities. (**E**) Total energy, demagnetizing energy, and Zeeman energy difference for anchored HEAs within 2 to 6 GHz through micromagnetic simulation. (**F**) Imaginary permeability values for anchored and isolated HEAs at 2 to 6 GHz. (**G**) In situ electron holography images and corresponding stray magnetic fields for nano-HEAs anchored on HEO nanosheets with increasing temperature from 300 to 900 K. (**H**) Variations of μ″ values for anchored and isolated HEA nanoparticles with increasing temperature from 300 to 900 K (applied frequency region: 2 to 6 GHz). Scale bars: (A) 10 nm and (inset) 50 nm, (B) 10 nm and (inset) 100 nm, (C) 10 nm and (inset) 50 nm, and (G) 100 nm.

The magnetic properties of the anchored high-entropy nanoalloys are elucidated through the nanoscale visualization of their magnetic domain structures. Using an off-axis magnetic holography technique, we observe that the anchored HEA nanoparticles exhibit well-defined sub–20-nm vortex magnetic domains (Fig. 3A). The effective formation of such nanoscale ordered vortex domains serves a pivotal role in enhancing magnetic properties of anchored nanoalloys. By contrast, the isolated HEA nanoparticles and anchored CrCo nanoalloys display relatively disordered magnetic nanostructures (Fig. 3B and fig. S39). The formation of a well-structured vortex-like magnetic configuration in the anchored high-entropy nanoalloys can be ascribed to two primary factors: (i) The synergistic effect of the high-entropy treatment and the interfacial entropy compensation strategy suppresses the surface effects and ensures a more uniform stress distribution within the anchored HEA nanoparticle, effectively mitigating the potential distribution of internal magnetic moments caused by abrupt stress variations and establishing a foundation for the development of a well-ordered and unified magnetic configuration; (ii) the notable stress field gradient that existed at the entropy-increased heterointerfaces surrounding the nanoalloy prompts the magnetic moments in the edge regions to align along these constructed interfaces to minimize energy, thereby generating a ring-like magnetic moment arrangement at the nanoparticle edges. Owing to the uniformity of the overall stress field, this magnetic moment alignment can propagate into the interior regions of nanoalloy and ultimately lead to the formation of a well-defined vortex domain that extends across the entire HEA nanoparticle. The configuration of the external magnetic stray field is also examined. As illustrated in Fig. 3C and fig. S40, a strong magnetic coupling of stray field is detected among anchored HEAs. By contrast, weak stray fields are observed in the isolated HEAs and anchored CrCo nanoalloys, resulting from the disordered internal magnetic configurations. With the argument about the weak magnetic signals of the HEO nanosheet substrate (fig. S41), it can be inferred that the aforementioned magnetic phenomenon primarily arises from the anchored HEA nanoalloys.

Furthermore, the macroscopic magnetic properties of the high-entropy system are investigated. Given the negligible magnetism of the HEO substrate, the subsequent analysis of magnetic characteristics focuses exclusively on the anchored HEA nanoparticles using rational methodologies to eliminate the influence of the weakly magnetic substrate (Supplementary Text and fig. S42). As shown in Fig. 3D, the analysis of hysteresis loops, measured via a vibrating sample magnetometer (VSM), reveals that HEAs surrounded by the entropy-enhanced heterointerfaces exhibit stronger intrinsic magnetism than the isolated HEAs. Specifically, the saturation magnetization (*M*_s_) increases by 80%, and the coercivity (*H*_c_) shows a 153% improvement, attributing to the presence of an ordered vortex magnetic configuration. Notably, this magnetic performance also surpasses that of anchored CrCo nanoalloys (fig. S43).

The response of the anchored HEA nanoparticles to an external stimulus of the high-frequency EM field is explored. Theoretical analysis of micromagnetic simulation reveals that, compared with the isolated HEA nanoparticles and a non–high-entropy system, the system with nano-HEAs anchored on HEO nanosheets exhibits a stronger demagnetizing effect and a higher total energy under the external magnetic field with similar strength (similar Zeeman energy), indicating its greater response to EM waves with various frequencies (Fig. 3E and fig. S44). Notably, a broad and enhanced peak is detected across a frequency range of 3.3 to 6.0 GHz, suggesting the pronounced resonance behavior of synthesized products to an external EM field within this frequency range.

To further elucidate the EM response experimentally, the frequency-dependent imaginary part of complex permeability (μ″, which typically reflects the magnetic loss capability of external EM waves) is measured using a network analyzer. As shown in Fig. 3F and fig. S45, a pronounced enhancement of μ″ is observed for anchored HEA nanoparticles, signifying their superior magnetic attenuation capability for high-frequency EM waves compared with the isolated HEA nanoparticles (135% improvement) and anchored CrCo nanoalloys (81% enhancement) (*35*). In addition, a broad natural resonance peak is observed at a frequency range that closely matches the simulated results. Overall, the prepared high-entropy nanoalloys anchored on HEO nanosheets exhibit enhanced intrinsic magnetism and EM response capability, attributed to the synergistic effect of the high-entropy state and interfacial entropy compensation.

Furthermore, the thermal stability of the magnetic characteristics is studied. The electron holography combined with the in situ heating module is used to explore the magnetism variation (Fig. 3G). Specifically, the anchored HEA nanoparticles retain the stable external stray field up to 700 K, graphically demonstrating their great magnetic stability over a wide temperature range. With a further increase in temperature, the external magnetic field starts to diminish and the sample transitions to a paramagnetic state (*36*). Owing to its great high-temperature resistance, the high-entropy system demonstrates a consistent μ″ value within the working temperature range (300 to 800 K), maintaining a value above 85% of its initial value at room temperature (Fig. 3H). In comparison to typical isolated HEA nanoparticles, this proposed system exhibits a slower decay trend and better retention of the magnetic properties, attributing to the temperature insensitivity of the regular vortex magnetic domains.

### Expanding applications of anchored high-entropy nanoalloy in EM wave modulation

With technological advancements and the widespread adoption of the internet of things and artificial intelligence interaction, the integration of the latest 5G wireless communication technology into human life continues to intensify, establishing its dominant position in daily activities. Meanwhile, the concomitant issue of EM pollution is becoming increasingly severe, posing considerable threats to human health and data transmission security. One effective approach to address this problem involves using EM absorbing materials to capture excessive external EM waves and subsequently dissipate them as thermal energy. Within the primary spectrum of the 5G technology (3.3 to 6.0 GHz), magnetic absorbers are particularly favored because of their pronounced magnetic resonances within this frequency range (*37*, *38*). However, current mainstream magnetic attenuation materials generally exhibit insufficient magnetic loss capabilities, as evidenced by their narrow effective absorption bandwidths and weak dissipation intensity. These insufficiencies limit their ability to meet the practical demands for contemporary EM pollution mitigation. In this study, because of the high-entropy treatment and the interfacial entropy compensation strategy, the reported high-entropy system demonstrates enhanced magnetism and magnetic loss capability compared with traditional ferromagnetic absorbers, showing potential for effectively tackling EM pollution within the 5G spectrum.

**Fig. 4.**
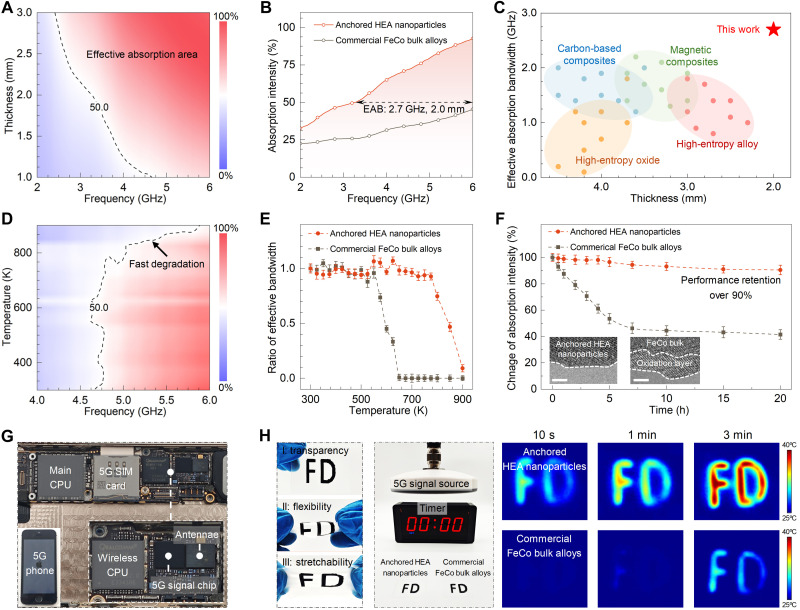
EM wave modulation of anchored HEA nanoparticles. (**A**) 2D color mapping of EM absorption intensity under room temperature with the frequency from 2 to 6 GHz and the thickness from 1 to 3 mm. (**B**) Calculated absorption intensity for anchored HEA nanoparticles and commercial FeCo bulk alloys under 2 mm. EAB, effective absorption bandwidth. (**C**) Comparison of the effective absorption bandwidth and corresponding thickness of various absorbers. (**D**) 2D absorption intensity mapping with temperature from 300 to 900 K in the frequency range of 2 to 6 GHz (thickness: 2 mm). (**E**) Variations of effective bandwidth for anchored HEA and commercial FeCo with increasing temperature from 300 to 900 K. (**F**) Change of absorption intensity for anchored HEA and commercial FeCo after thermal treatment in air at 600 K with various times. The inset images show the lattice at the edge of anchored HEA and commercial FeCo after thermal treatment. h, hours. (**G**) Digital photos of wireless communication modules in a 5G smartphone. CPU, central processing unit; SIM, subscriber identity module. (**H**) Digital photos of the flexible EM absorbing device and thermal infrared images of devices composed of anchored HEA and commercial FeCo bulk alloys under the irradiation of a 5G signal source with different durations. Scale bars: [inset in (F)] 2 nm.

To verify this potential, the EM attenuation performance of the proposed system at room temperature is systematically evaluated. As depicted in the thickness- and frequency-dependent plots of the EM dissipation intensity (Fig. 4A and fig. S46), the anchored HEA nanoparticles demonstrate a considerable enhancement of EM absorption capability compared with the commercial FeCo bulk alloys, isolated HEA nanoparticles, and anchored CrCo nanoalloys, while the HEO nanosheets alone contribute negligibly to EM dissipation. A detailed analysis of the EM parameters (figs. S47 and S48) attributes this improvement to the increased permeability and magnetic loss capabilities of the anchored HEAs, caused by the formation of stable vortex magnetic domains and intense magnetic coupling. At a standard thickness of 2.0 mm, which is typical for commercial applications, the anchored HEAs exhibit a broad effective absorption bandwidth (where the dissipation intensity exceeds 50%) from 3.3 to 6.0 GHz, covering almost the entire standard 5G frequency spectrum that the traditional EM absorbers cannot achieve (Fig. 4B). Moreover, in comparison with other dominant EM materials, including carbon-based composites, magnetic alloys, HEOs, and high-entropy ceramics, the prepared high-entropy system demonstrates a superior effective absorption bandwidth and a thinner coating thickness (Fig. 4C and table S2) (*39*–*74*).

Given the extreme conditions required in the practical applications of EM absorption materials, the resistance of attenuation performance to high-temperature and long-term oxidation is explored. As described in Fig. 4 (D and E), with an increasing test temperature up to ~800 K, the effective absorption bandwidth of the anchored HEA nanoparticles remains nearly unchanged. Afterward, rapid degradation is observed, caused by the transformation of magnetic configuration from the vortex to the paramagnetic state. In contrast, for commercial FeCo bulk alloys, the rapid deterioration in EM dissipation capability is advanced to about 600 K (fig. S49). This phenomenon highlights the excellent thermal stability of EM response for the high-entropy system. Chemical stability is then examined by measuring the variation of EM attenuation ability following heating treatment at 600 K in the atmosphere for different durations. As shown in Fig. 4F, the EM absorption intensity in the anchored high-entropy nanoalloys is maintained at more than 90% after a long-term oxidation treatment for 20 hours, while the performance of the commercial FeCo absorbers instantly declined to 40% after a short heating process for solely 5 hours. In addition, after thermal treatment, a distinct oxidation layer is observed on the commercial FeCo bulk alloys, whereas no oxidation phenomenon is detected on the anchored HEA nanoparticles (insets in Fig. 4F and fig. S50). Overall, the fabricated high-entropy system exhibits superior thermal resistance and antioxidation capability in comparison to conventional ferromagnetic absorbers.

Furthermore, the practical application of the fabricated composites as functional EM devices is studied. In daily life, 5G signals predominantly originate from the wireless communication modules integrated into smartphones (Fig. 4G). Therefore, a highly flexible and stretchable 5G signal absorbing device is fabricated via printing the anchored HEA nanoparticles onto a transparent polydimethylsiloxane substrate (Fig. 4H). In addition, compared with a counterpart device composed of commercial FeCo bulk alloys, this device exhibits superior performance in harnessing external EM energy within the 5G frequency spectrum and subsequentially effectively converting it into thermal energy.

## DISCUSSION

This work proposes an advanced strategy for the in situ growth of the HEA nanoparticles on the ultrathin HEO nanosheets to construct abundant entropy-increased heterointerfaces surrounding the magnetic nanoalloys. The synergistic effect of the high-entropy treatment and interfacial entropy compensation strategy simultaneously enhances the internal stress field of the precipitated HEAs while suppressing lattice distortions and defects at the edges. This facilitates a more uniform distribution of the stress field throughout the nanoalloys, thereby reducing the resistance to the establishment of an ordered magnetic configuration. In addition, the magnetic moments tend to align preferentially along the heterogeneous interfaces. Owing to the relatively homogeneous stress distribution, such a ring-like arrangement of magnetic moments would propagate throughout the entire nanoparticle and eventually form stable sub–20-nm vortex magnetic domains. Collectively, these effects remarkably suppress the surface effect within the anchored HEA nanoparticles, resulting in magnetic properties being superior to the isolated HEA nanoparticles. Regarding the EM response capability, the system with nano-HEAs anchored on HEO nanosheets exhibits remarkable attenuation intensity and an extended effective absorption bandwidth within the 5G standard frequency range, far outperforming traditional FeCo absorbers. Moreover, because of the formation of stable vortex magnetic configuration and strong interfacial interaction, the substantially improved thermal and chemical stabilities of EM dissipation capability are further demonstrated, surpassing commercial ferromagnetic alloys. These superior characteristics render the developed high-entropy system promising candidates for advanced applications in next-generation EM modulation, magnetic storage and computing, and biomedical imaging and drug delivery.

## MATERIALS AND METHODS

### Synthesis of 2D HEO nanosheets

The HEO nanosheets were prepared through the modified self-template method (*75*, *76*). Typically, equimolar amounts of metal salts, 1.001 g of chromium(III) nitrate nonahydrate, 0.628 g of manganese(II) nitrate tetrahydrate, 1.011 g of iron(III) nitrate nonahydrate, 0.728 g of cobalt(II) nitrate hexahydrate, and 0.727 g of nickel(II) nitrate hexahydrate, were dissolved in 20 ml of deionized H_2_O. Then, 4 g of PVP was added into the solution under stirring with a magnetic agitator for 2 hours. After metal salts were fully absorbed by PVP molecules, a rapid freezing procedure was used by dripping uniform solution into the liquid nitrogen and subsequently freeze-drying for 48 hours to completely remove H_2_O. Last, during the subsequent foaming procedure, the prepared mixture was annealed in a muffle furnace at 500°C for 2 hours with a heating rate of 2°C min^−1^. During this process, nitrates decomposed at high temperatures, releasing gases that drove the molten PVP to form nanolayered structures. These PVP nanosheets adhered to the newly formed oxide nanoparticles and subsequently decomposed under high-temperature conditions, ultimately yielding HEO nanosheet products. The preparation of contrast (Cr_1/2_Co_1/2_)_3_O_4_ nanosheets was similar, except that only two metal salts, 2.501 g of chromium(III) nitrate nonahydrate and 1.819 g of cobalt(II) nitrate hexahydrate, were used.

### Synthesis of anchored HEA nanoparticles

The anchored HEA nanoparticles were fabricated via a one-step reduced treatment of HEO nanosheets. In detail, HEO precursors were placed in a ceramic crucible without a covering, ensuring the adequate contact with the reducing gas. Under the 5 vol% H_2_/Ar atmosphere, powders were reduced in a tubular furnace with a slow heating rate of 1°C min^−1^. When the temperature reached 500°C, the heating program stopped immediately and then cooled naturally to obtain the high-entropy system with nano-HEAs anchored on HEO nanosheets. In exploring the size of precipitated HEA nanoparticles, the maximum reduction temperature was adjusted to 400°, 600°, 700°, and 800°C. In exploring the generalization, the binary (2: CrCo), quaternary (4: CrFeCoNi), and senary (6: CrMnFeCoNiCu) systems were synthesized by just adjusting the usage of corresponding nitrate salts while keeping the unchanged total molar amount.

### Synthesis of isolated HEA nanoparticles

The isolated HEA nanoparticles were synthesized through a rapid reduction reaction in solution. Specifically, 2.994 g of chromium(III) nitrate nonahydrate, 1.014 g of manganese(II) sulfate monohydrate, 3.336 g of iron(II) sulfate heptahydrate, 5.060 g of cobalt(II) sulfate heptahydrate, and 4.731 g of nickel(II) sulfate hexahydrate were added into 100 ml of H_2_O to mix a uniform solution. Then, under a water bath of 45°C and mechanical stirring, another well-mixed solution, containing 22 g of sodium hydroxide, 45 ml of hydrazine hydrate aqueous solution, and 20 ml of H_2_O, was slowly dropped into the above solution. After stirring for 2 hours, the black precipitates were obtained by centrifugation at 4000 rpm, washed with water and ethanol, and dried at 60°C overnight.

### Materials characterization

The morphology and atomic arrangement were measured via a field-emission scanning electron microscope (Hitachi, S-4800, 5 kV), a transmission electron microscope (JEOL, JEM-2100F, 200 kV), and a dual spherical aberration-corrected transmission electron microscope (FEI, Titan Themis Z, 300 kV). The thickness of the nanosheet precursor was measured by AFM (Bruker Dimension Edge). The crystal structure was examined using an x-ray diffractometer (Bruker, D8-Advance) with Ni-filtered Cu Ka radiation (2θ range, 10° to 85°; scan rate, 2°/min; step size, 0.01), while Ag radiation was used for the measurement of atomic PDF pattern (2θ range, 5° to 145°; scan rate, 0.5°/min; step size, 0.02). The exploration of chemical composition was characterized by XPS performed on an ESCALAB MKII x-ray photoelectron spectrometer (Al K_α_, *hv* = 1486.6 eV). The magnetic property was explored through the superconducting quantum interference device magnetometer (MPMS VSM, Quantum Design Company of USA), and the visualization of magnetic domains and stray field was achieved through off-axis electron holography using the modified TEM system (JEOL, JEM-2100F, 200 kV) with an in situ heating procedure from 300 to 800 K.

### Characterization of internal magnetic configuration and external stray fields at the nanoscale

Off-axis electron holography was performed here to probe the internal magnetic configuration for magnetic samples at the nanoscale. Initially, two principal electron holograms were captured: one featuring the sample under analysis and the other representing only the vacuum. The magnetic signals were extracted by computing the differential between these two holograms. Following Fourier transform, noise selection, and inverse Fourier transform procedures, a spatial distribution map of the internal magnetic moments was constructed. To mitigate spurious signals induced by sample thickness, two electron holograms were obtained and addressed from the front and rear of the sample at the same location. For the examination of external stray fields, off-axis electron holography was applied again to capture electron holograms at a relatively low resolution.

For the characterization of magnetic configurations with in situ heating, a Lorentz transmission electron microscope was used as the central instrument, coupled with an advanced in situ heating module. The GATAN in situ sample holder can heat the characterized sample by applying a certain voltage to the sample holder, and the heating resistor starts to work. In addition, the temperature sensor at the front of the sample holder provided real-time measurements of the actual temperature near the sample. To monitor the internal sample signals, the InfiniiVision DSOX6002A oscilloscope was used, ensuring the accurate capture of experimental data. In investigating the stability of stray fields, a ramping program, from 300 to 900 K, in steps of 100 K, was implemented. At each temperature point, an electron hologram was acquired after the target temperature had equilibrated over a sufficient stabilization period.

### Measurement of EM absorption performance

For the exploration of EM dissipation capability, a network analyzer (Keysight, N5230C) was used. For the measurement at room temperature, the tested powders were adequately mixed with a certain amount of molten paraffin and pressed into cylindrical rings with standard inner and outer diameters of 3.00 and 7.00 mm, respectively. Using the single coaxial line method, the frequency-dependent EM parameters were collected. For the evaluation of EM attenuation performance at high temperature, the waveguide method was used. The samples were blended uniformly with polyvinyl alcohol (the filling ratio was identical to that tested under room temperature) and subsequently shaped into a rectangular block. The tested temperature was set from 300 to 900 K and maintained for 30 min at each temperature point. The frequency-dependent EM parameters, complex permeability (μ_*r*_ = μ′ − *j*μ″) and permittivity (ɛ_*r*_ = ɛ′ − *j*ɛ″), were determined using a network analyzer. In general, the real parts of complex permeability and permittivity (μ′ and ɛ′) correspond to the storage capability of external EM energy, while the imaginary parts (μ″ and ɛ″) reflect the lossy capability of stored energy. For the assessment of EM absorption capability, the frequency-dependent reflection loss (*RL*) value is calculated on the basis of the coaxial theory (*77*–*79*)

$$Z_{\text{in}}=Z_{0}\sqrt{\mu_{r}/\varepsilon_{r}}\text{tanh}\left[j\left(2\pi fd/c\right)\sqrt{\mu_{r}\varepsilon_{r}}\right]$$

$$\text{RL}\left(\text{dB}\right)=20\text{log}\left[\left(Z_{\text{in}}-Z_{0}\right)/\left(Z_{\text{in}}+Z_{0}\right)\right]$$

where *Z*_in_ and *Z*_0_ are the impedance values of the tested absorber and free space, while μ_r_ and ɛ_r_ are the EM parameters of complex permittivity and permeability, respectively. *j* is the imaginary unit, *f* denotes the frequency of incident EM waves, *d* is the corresponding thickness of the absorbing layer, and *c* is the propagation velocity of EM waves.

On the basis of the obtained RL value, the calculation of EM absorption intensity (*A*%) was shown as follows (*80*–*82*)

$$A\%=\left(1-\frac{10^{-\frac{\text{RL}}{10}}}{1+10^{-\frac{\text{RL}}{10}}}\right)\times 100\%$$

This parameter represented the dissipation capacity of the absorber toward EM waves.

Furthermore, to distinguish between the contributions of magnetic and dielectric losses, the magnetic tangent value (tanμ) and dielectric tangent value (tanɛ) were used (*83*–*85*)

$$\text{tanμ}=\mu^{\prime \prime }/\mu^{\prime }$$

$$\text{tanε}=\varepsilon^{\prime \prime }/\varepsilon^{\prime }$$

The relative magnitudes of these two parameters offered insight into the predominant role of either magnetic or dielectric loss in the overall EM attenuation behavior.
