# Supplementary material for: High-entropy nanoalloys anchored on entropy-compensating two-dimensional oxides for enhanced nanomagnetism
Source: Sci Adv. 2025 Nov 21;11(47):eadv8411. doi: 10.1126/sciadv.adv8411 (PMC12637284; doi:10.1126/sciadv.adv8411)
Supplement: Supplementary file 1 — Supplementary Text Figs. S1 to S50 Tables S1 and S2 [file sciadv.adv8411_sm.pdf]

Science Advances
AAAS

Supplementary Materials for

High-entropy nanoalloys anchored on entropy-compensating two-dimensional oxides for enhanced nanomagnetism

Xiaodi Zhou *et al.*

Corresponding author: Renchao Che, rcche@fudan.edu.cn; Hualiang Lv, lv_hl@fudan.edu.cn

Sci. Adv. **11**, eadv8411 (2025)
DOI: 10.1126/sciadv.adv8411

This PDF file includes:

Supplementary Text
Figs. S1 to S50
Tables S1 and S2

Supplementary Text

The method of removing the interference of HEO substrate in comparing magnetic properties and electromagnetic responses

For the system that nano HEAs anchored on HEO nanosheets, given the negligible magnetism of HEO substrate, this study concentrates on the magnetic behaviors of the precipitated HEA nanoparticles. However, the weight of the sample remains a crucial factor in most magnetic characterizations. To facilitate a more accurate comparison of the magnetic properties of HEA nanoparticles anchored on HEO nanosheets, here we employ several methods to eliminate the contribution of the weakly magnetic HEO substrates to the overall sample weight. To exclude the effect of HEO substrate, the phase content must be determined first. In this context, the thermogravimetric (TG) analysis is implemented to quantify the content of HEA nanoparticles in the prepared system. As illustrated in **fig. S42**, after the thorough oxidation of the prepared high-entropy system, the mass increases by 17 wt.% compared to the initial mass. Following TG analysis, the XRD pattern reveals the presence of only the spinel oxide phase, indicating that the mass change arises from the conversion of the precipitated HEA nanoparticles into the oxide phase. Therefore, the content of HEA phase in this high-entropy system can be approximated according to following chemical reaction equation and formula (noting that the relative atomic mass of Fe lies in the middle of five metal elements and the molar content of Fe is close to 1/5 in both the HEO and HEA phases, thus we use Fe as a surrogate for all five metal elements):

$$3Fe + 4O \rightarrow Fe_3O_4 \qquad (S1)$$

$$\text{Content of HEA} = \frac{0.17\times m_0\times\frac{3}{4}\times\frac{56}{16}}{m_0} = 0.446 \qquad (S2)$$

where m_0 denotes the initial mass. For simplicity, it is assumed that the weight fraction of precipitated HEA nanoparticles in the fabricated high-entropy system is approximately 50 wt.%.

i) Characterization of hysteresis loops

The weight of the tested sample must be considered when characterizing hysteresis loops. For the calculation of hysteresis loop for nano HEAs anchored on HEO nanosheets, the weight contribution of HEO substrate is neglected. This operation is justified by the negligible saturation magnetization of pure HEO nanosheets, which renders their contribution inconsequential (**figs. S41** and **S43**).

ii) Permeability and electromagnetic attenuation performance

The permeability and electromagnetic attenuation performance are assessed using a network analyzer. During the measurement, the tested sample is mixed with a specific amount of paraffin or polyvinyl alcohol. To eliminate the influence of the oxide substrate, the specific mass percentages are set as 80 wt.% for HEAs anchored on HEO nanosheets, binary system (CrCo) and pure HEO nanosheets, and 40 wt.% for isolated HEA nanoparticles and commercial FeCo nanoflakes. This approach is validated by the weak permeability and poor electromagnetic absorption performance of pure HEO nanosheets (**figs. S45-46**).

Supplementary Figures

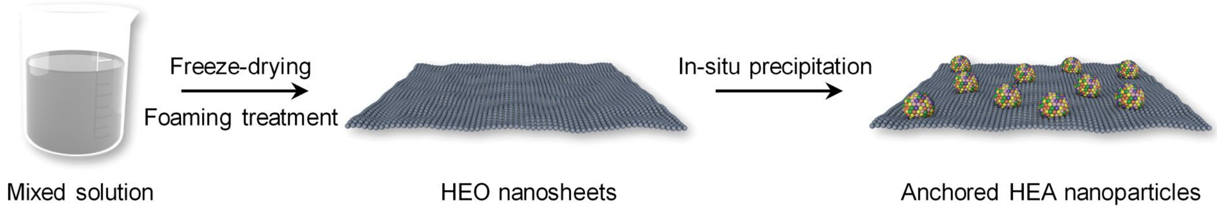

Fig. S1. Schematic illustration of the synthesis process for the high-entropy system that nano HEAs anchored on two-dimensional HEO nanosheets. Firstly, metal salts and polyvinylpyrrolidone are dissolved into deionized water to form a uniform solution. After the freeze-drying and foaming treatment, two-dimensional HEO nanosheets are collected. Following with the in-situ thermal reduction treatment, the high-entropy system that nano HEAs anchored on HEO nanosheets is obtained.

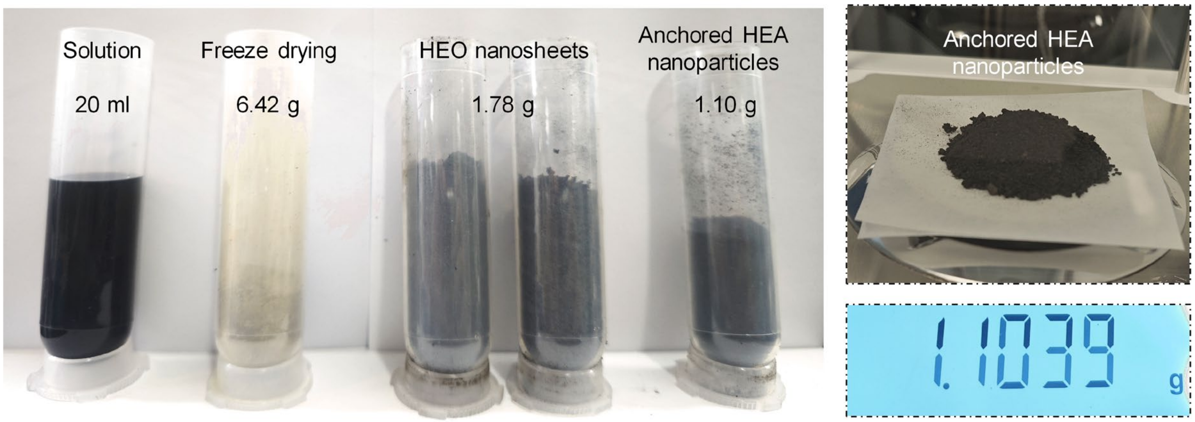

Fig. S2. Photographic images of samples at each procedure. Due to the uncomplicated freeze-drying and sintering treatments, it can produce more than one gram of sample per batch, enabling the possibility of future mass production.

A B C

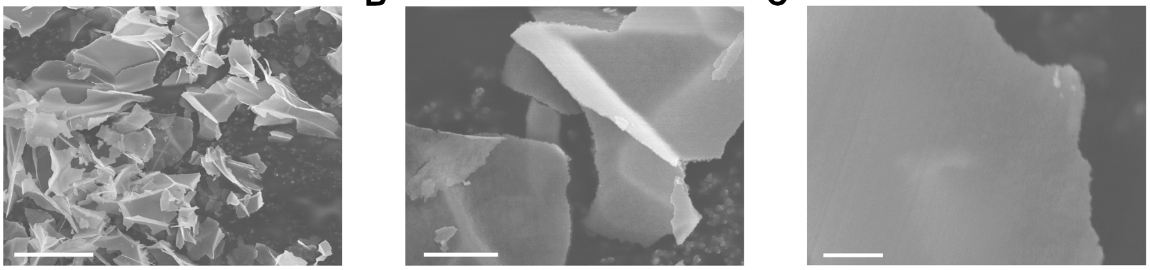

Fig. S3. Scanning electron micrograph for HEO nanosheets. Under various magnifications, the ultrathin lamellar morphology is confirmed. Scale bars: (A) 5 μm, (B) 1 μm, (C) 200 nm.

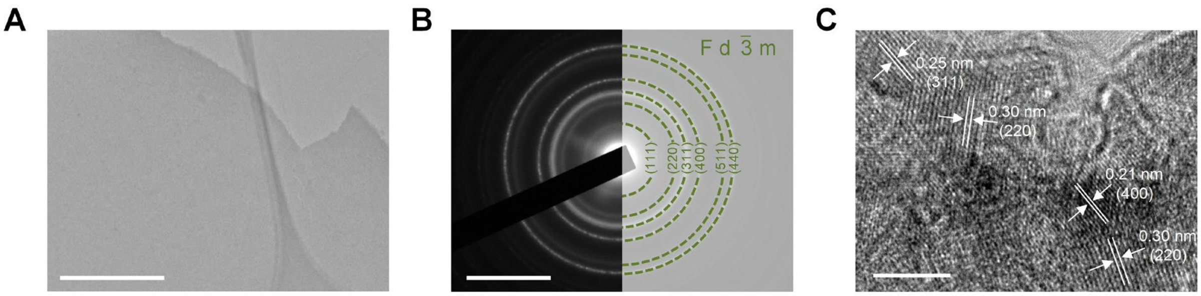

Fig. S4. The crystalline information of HEO nanosheets characterized via the transmission electron microscopy (TEM). (A) The TEM image, (B) the corresponding selected area electron diffraction (SAED) pattern, (C) the high-resolution TEM image showing lattice fringing. The prepared nanosheets exhibit a crystal phase corresponding to typical spinel oxide with a group space ($F\,d\,\bar{3}\,m$). Scale bars: (A) 500 nm, (B) 5 1/nm, (C) 5 nm.

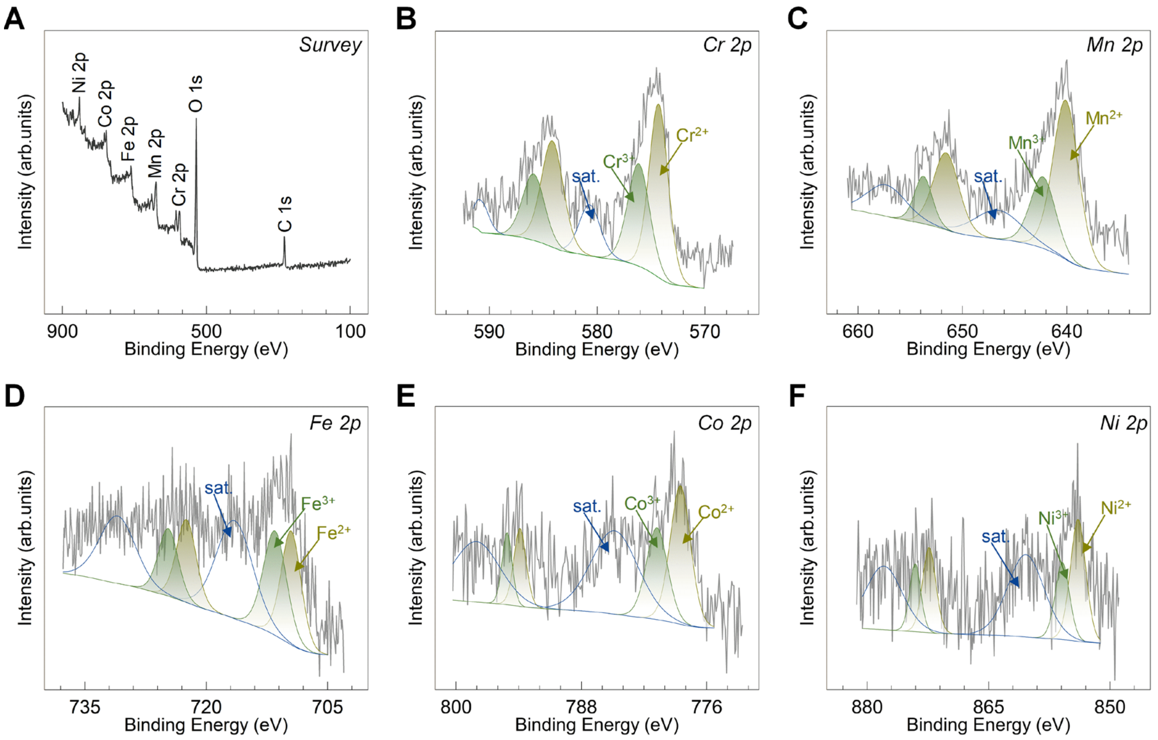

Fig. S5. Analysis of X-ray photoelectron spectroscopy for HEO nanosheets. (A) Survey spectrum, (B) Cr *2p*, (C) Mn *2p*, (D) Fe *2p*, (E) Co *2p*, (F) Ni *2p*. All metal elements exhibit divalent and trivalent states.

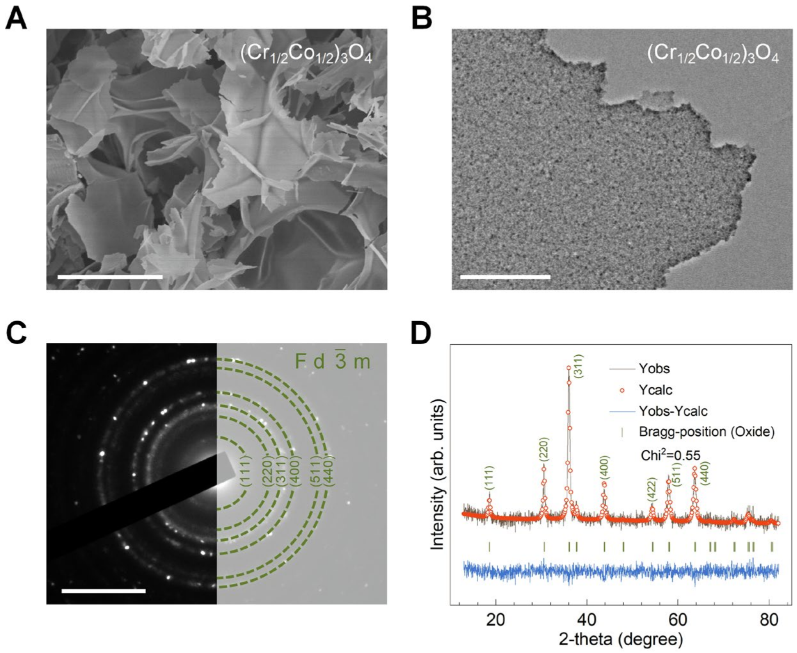

Fig. S6. The morphology and crystal characterization of $(Cr_{1/2}Co_{1/2})_3O_4$ nanosheets. This sample exhibits the thin nanosheet structure and typical spinel oxide phase. Scale bars: (A) 5 µm, (B) 200 nm, (C) 5 1/nm.

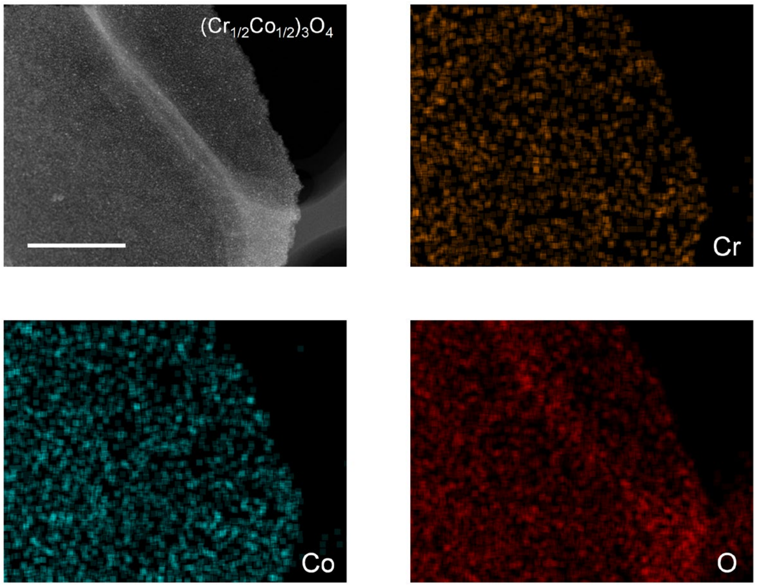

Fig. S7. The energy-dispersive X-ray spectroscopy (EDS) mapping of $(Cr_{1/2}Co_{1/2})_3O_4$ nanosheets. The elements of Cr, Co, and O are uniformly distributed within nanosheets, without any elemental separation. Scale bar: 500 nm.

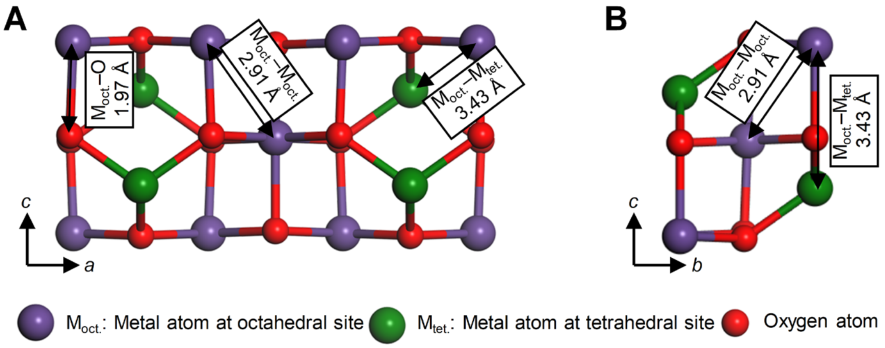

Fig. S8. The illustration of atom pairs in HEO crystal structure along (A) *b* axis and (B) *a* axis. This diagram exhibits the corresponding atom pairs corresponding to characteristic peaks in pair distribution function analysis for HEO nanosheets.

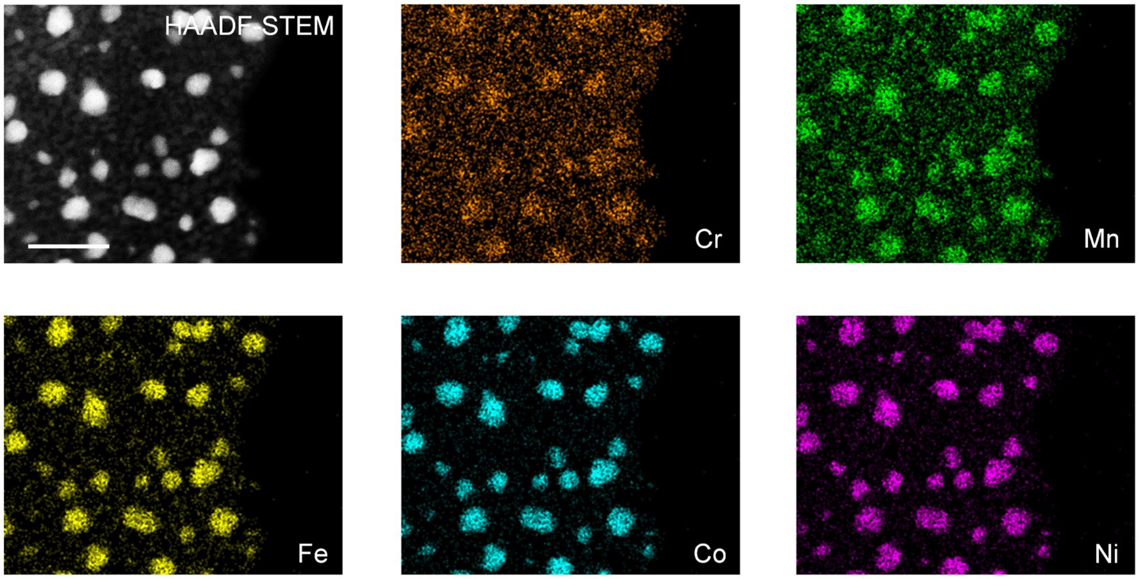

Fig. S9. The high-resolution energy-dispersive X-ray spectroscopy (EDS) mapping after the in-situ precipitation treatment of the HEO nanosheet precursor. The precipitated nanoparticles contain all metal elements, including Cr, Mn, Fe, Co and Ni. Scale bar: 50 nm.

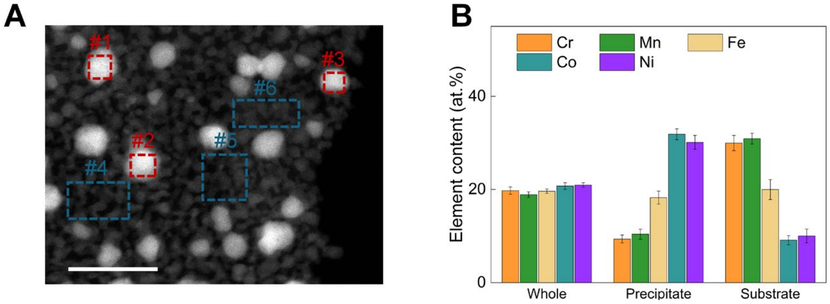

Fig. S10. Analysis of element content for precipitated and substrate phases based on the selected energy-dispersive X-ray spectroscopy (EDS) mapping. (A) Selected areas for the precipitate and substrate within the high-entropy system, (B) element content for the whole system, the precipitate and the substrate. For precipitated nanoparticles, the contents of Fe, Co, and Ni are more than Cr and Mn, with the elemental distribution in the substrate showing the reverse pattern.

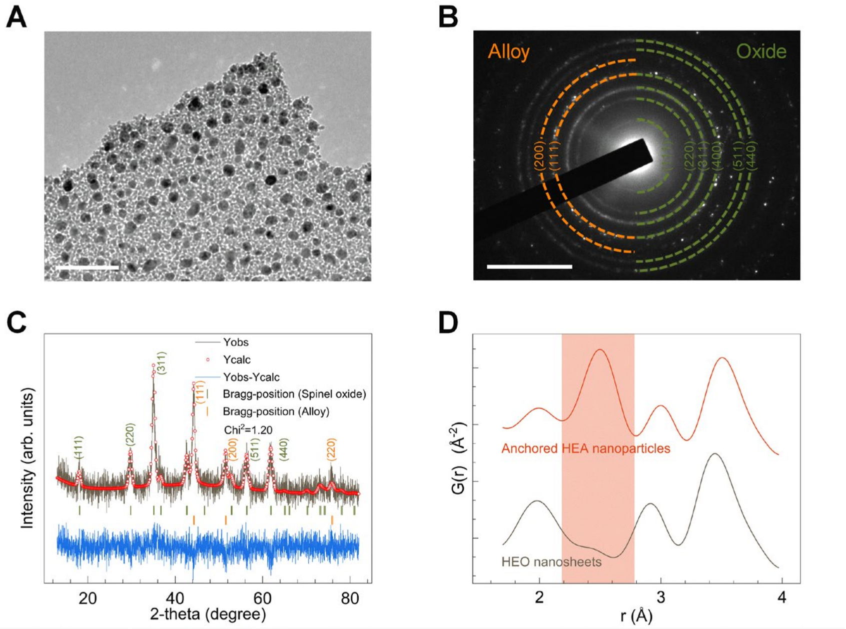

Fig. S11. The characterization of crystal structure for the high-entropy system that nano HEAs anchored on HEO nanosheets. (A) TEM image, (B) the corresponding SAED pattern, (C) Rietveld refinement of XRD pattern, (D) the pair distribution function (PDF) analysis. Two types of crystal structure are demonstrated, closing to typical spinel oxide and alloy phases. In the analysis of interatomic distance, compared with HEO nanosheets, a characteristic peak at 2.50 Å appears, referring to the distance of metal atoms within face-centered cubic (*fcc*) alloy. Additionally, the peak of oxide phase is slightly shifted to the right after the reducing treatment, might be attributed to the fact that more atoms with small diameter (Fe, Co, Ni) are precipitated than atoms of large size (Cr, Mn) in the oxide substrate, leading to the expansion of crystal cells and increase of atomic distance within the HEO substrate. Scale bars: (A) 100 nm, (B) 5 1/nm.

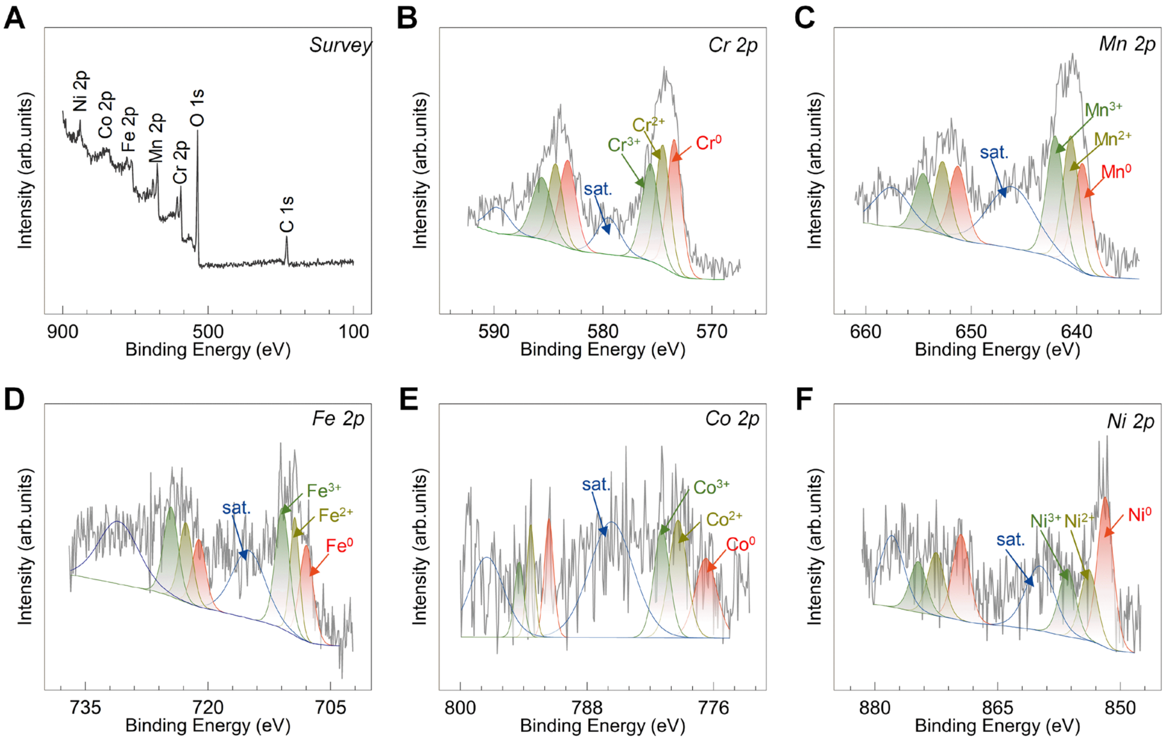

Fig. S12. Analysis of X-ray photoelectron spectroscopy for the high-entropy system that nano HEAs anchored on HEO nanosheets. (A) Survey spectrum, (B) Cr *2p*, (C) Mn *2p*, (D) Fe *2p*, (E) Co *2p*, (F) Ni *2p*. Except for the divalent and trivalent states, all metal elements exhibit characteristic peaks for the metallic state.

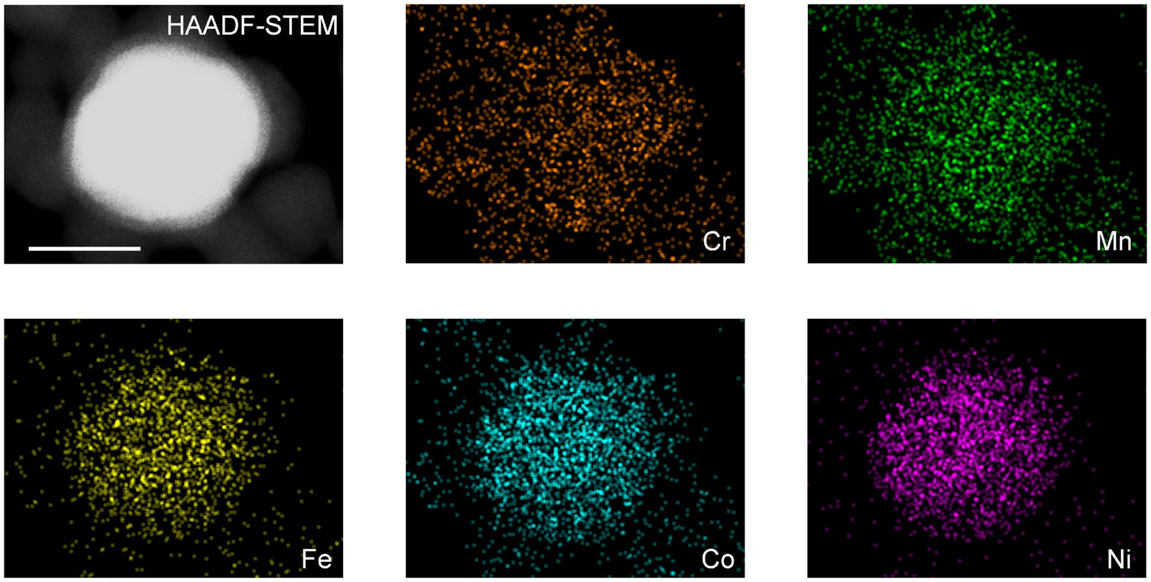

Fig. S13. The high-resolution energy-dispersive X-ray spectroscopy (EDS) mapping for a single precipitated HEA nanoparticle on HEO substrate. The HEA nanoparticle contains all metal elements, including Cr, Mn, Fe, Co and Ni. Scale bar: 10 nm.

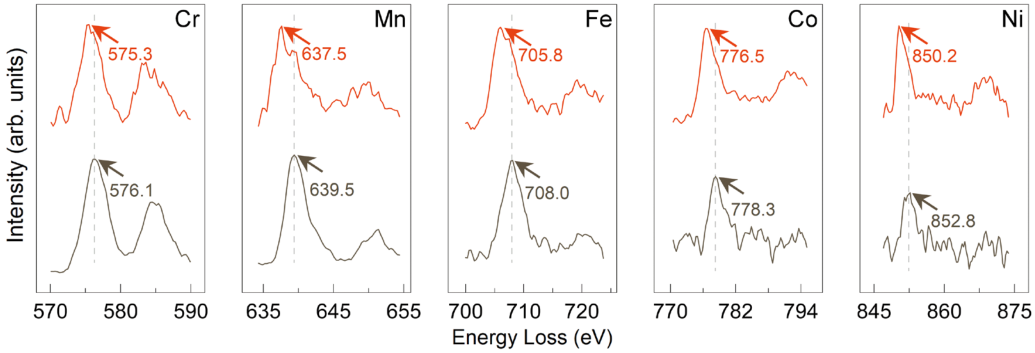

Fig. S14. The high-resolution electron energy loss spectroscopy (EELS) curves for each metal element. The red curve represents the precipitated phase, while the gray curve represents the bulk substrate. The characteristic peak of the precipitated nanoparticle exhibits a left shift in comparison to the bulk substrate, indicating the metallic state of Cr, Mn, Fe, Co and Ni in the nanoparticle.

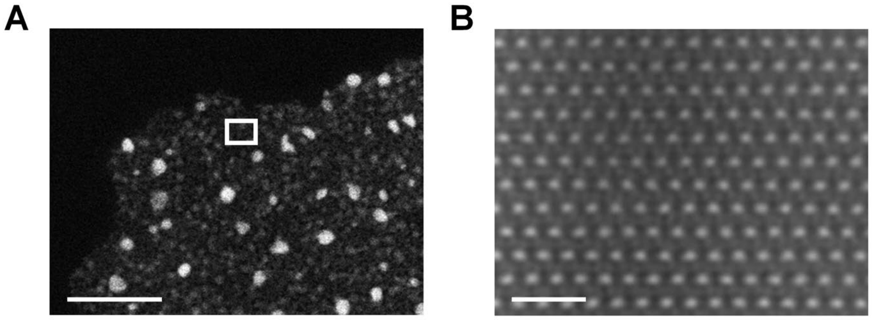

Fig. S15. The atomic HAADF-STEM image of the nanosheet substrate. (A) Low-resolution, (B) atomic resolution. The atomic arrangement of the substrate, viewed down the [102] zone axis, is consistent with the typical spinel oxide lattice. Scale bars: (A) 10 nm, (B) 1 nm.

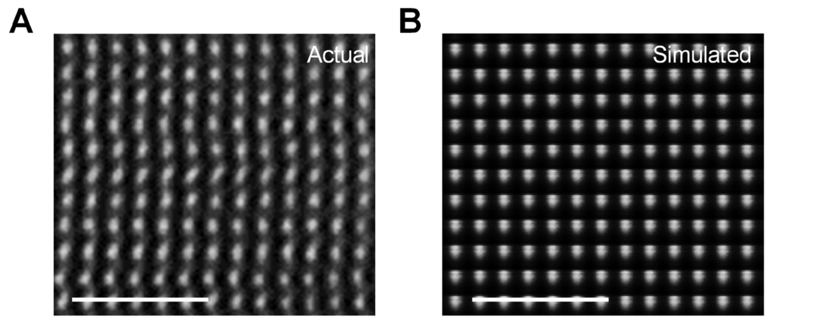

Fig. S16. The simulation of high-angle annular dark-field scanning transmission electron microscopy (HAADF-STEM) by quantitative scanning transmission electron microscopy (QSTEM) for precipitated HEA nanoparticles. (A) The actual HAADF-STEM image, (B) the simulated image. All atoms occupy lattice sites instead of interatomic space, excluding the formation of intermetallic compounds. Scale bar: 1 nm.

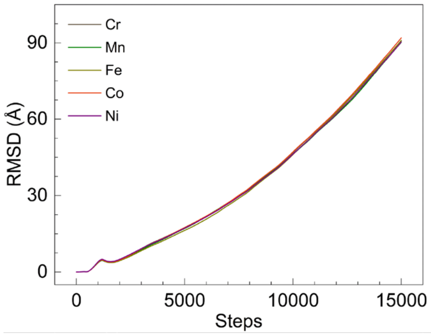

Fig. S17. The molecular dynamic simulation for the precipitation process of HEA nanoparticles at 500 ºC. The curves of root-mean-square displacement (RMSD) of five metal elements are approximate to each other without any obvious separation for 15000 steps, resulting in the homogeneous elemental distribution in precipitated HEA nanoparticles.

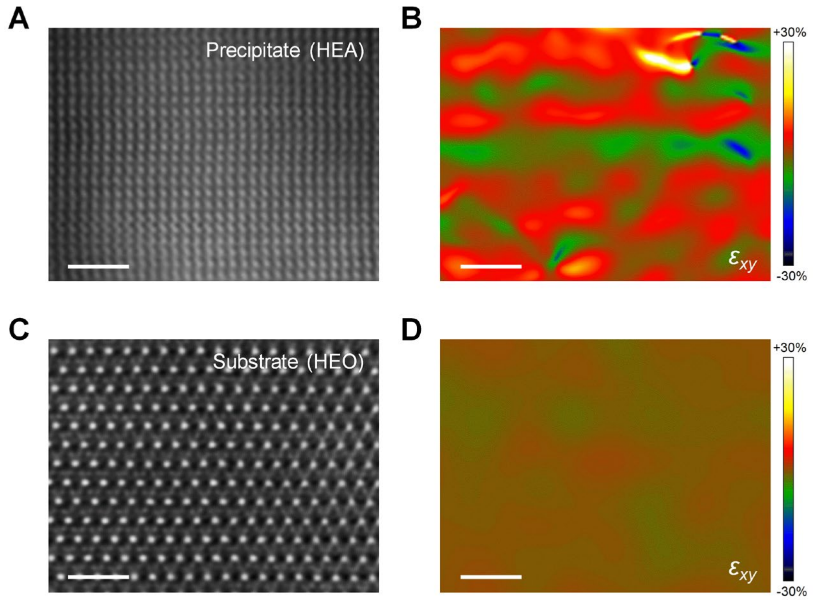

Fig. S18. Analysis of strain fields ε_{xy} via GPA method for precipitated HEA and HEO substrate in the high-entropy system that nano HEAs anchored on HEO nanosheets. (A, B) Precipitated HEA, (C, D) HEO substrate. The stress field in the precipitated nanoparticle is markedly greater than that in substrate, indicating the more severe lattice distortions in HEA nanoparticles. Scale bar: 1 nm.

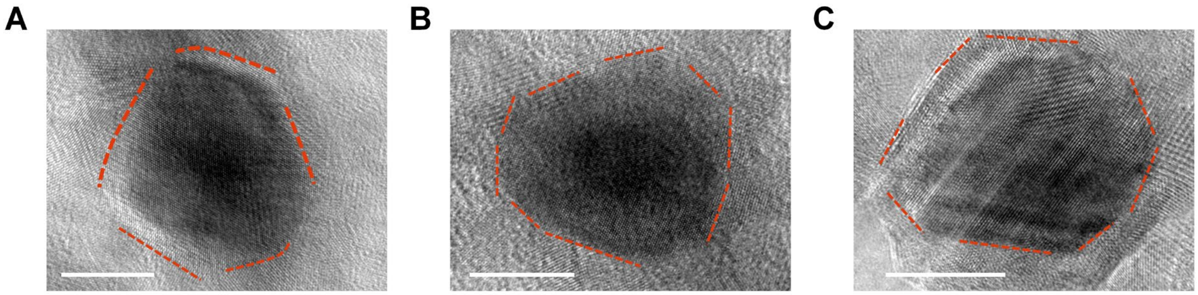

Fig. S19. The high-resolution TEM image for anchored HEA nanoparticles at various regions. The marked red dashed lines are heterointerfaces. Numerous heterointerfaces between precipitated HEA nanoparticles and HEO substrates are formed at the edge of HEA nanoparticles. Scale bar: 10 nm.

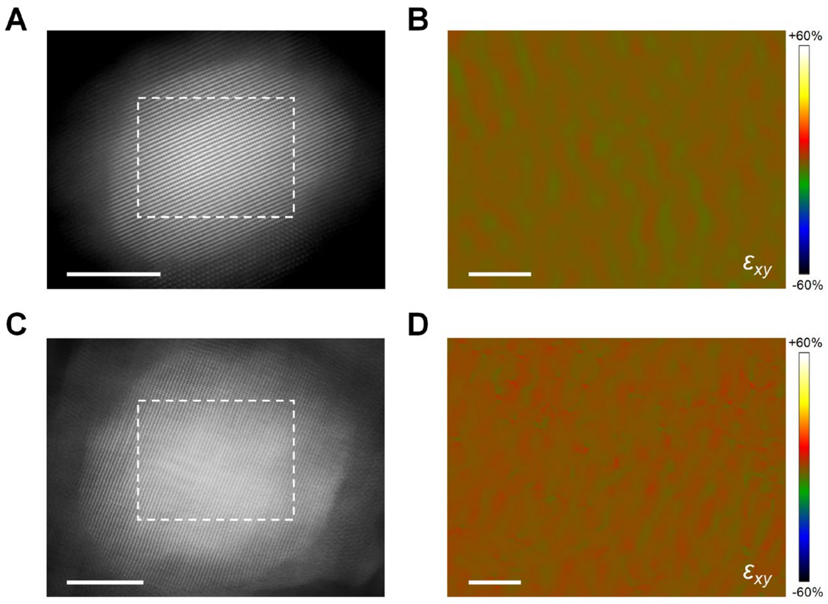

Fig. S20. The atomic HAADF-STEM images for anchored HEA nanoparticles at various regions and corresponding analysis of strain fields ε_{xy} via GPA method. The selected areas of strain analysis are marked as white dashed boxes. These anchored HEAs exhibit homogeneous stress distribution. Scale bars: (A, C) 5 nm, (B, D) 2 nm.

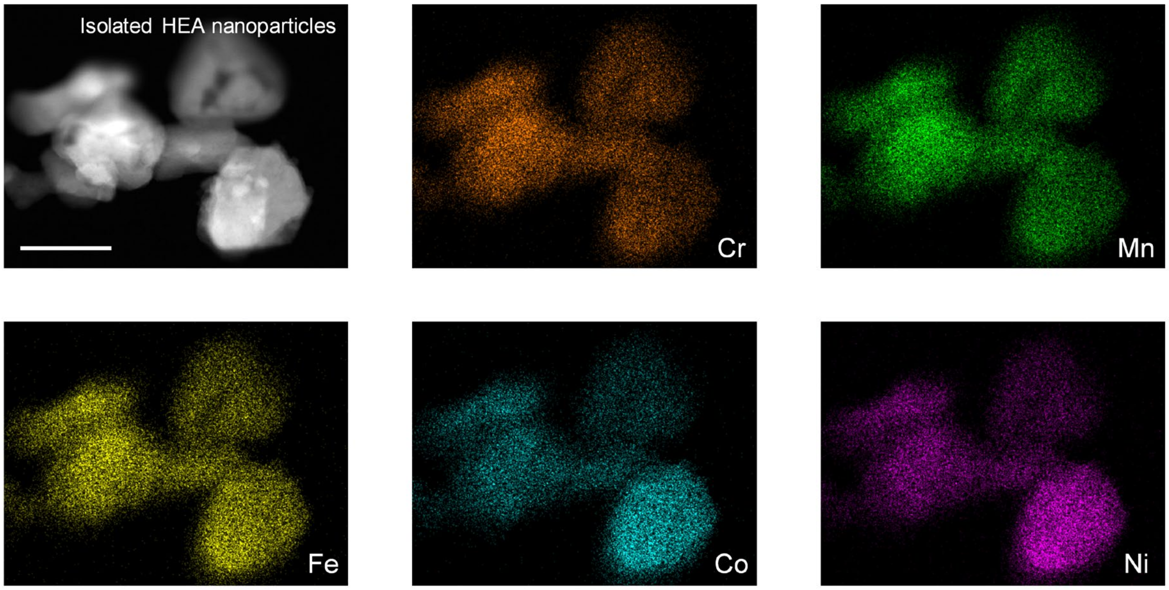

Fig. S21. The energy-dispersive X-ray spectroscopy (EDS) mapping of isolated HEA nanoparticles. The elements of Cr, Mn, Fe, Co, and Ni are uniformly distributed within nanoparticles, without any elemental separation. Scale bar: 20 nm.

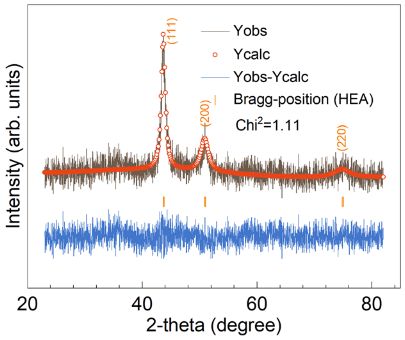

Fig. S22. Rietveld refinement XRD pattern of isolated HEA nanoparticles. Only one *fcc* crystal phase is detected, identical to the crystal structure of the anchored HEAs within prepared high-entropy system. The broadening of characteristic peaks could be attributed to the particle size on the nanoscale.

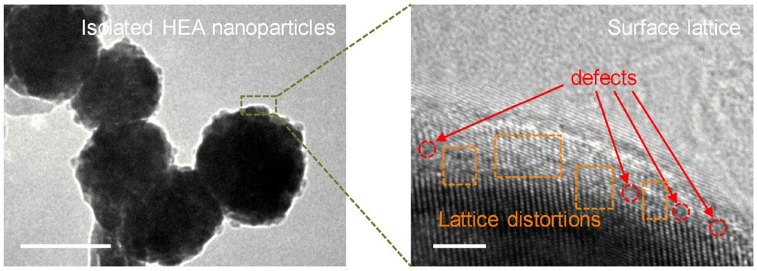

Fig. S23. The high-resolution TEM image for isolated HEA nanoparticles near the edge. It is obvious that a substantial amount of lattice distortions and defects exist at the edges. Scale bars: (Left) 20 nm, (Right) 2 nm.

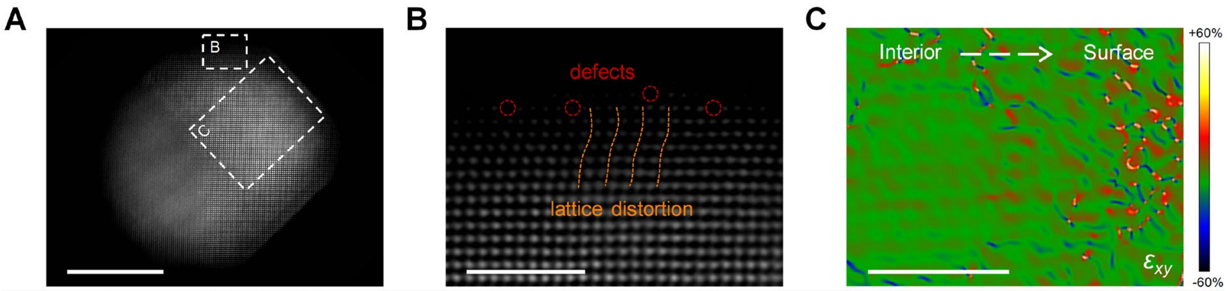

Fig. S24. The atomic HAADF-STEM image for isolated HEA nanoparticles and corresponding analysis of strain fields ε_{xy} via GPA method. For isolated HEAs, lattice distortions and defects near the edge region are detected at the atomic resolution. In addition, compared to the interior region, numerous abrupt stress variations exist near the nanoparticle's surface. Scale bars: (A) 10 nm, (B) 2 nm, (C) 5 nm.

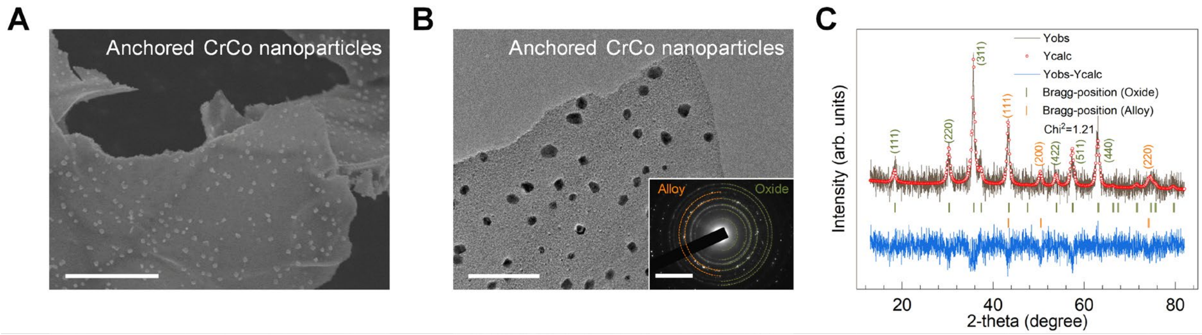

Fig. S25. The morphology and crystal characterization of the binary system (anchored CrCo nanoparticles). This system exhibits a thin nanosheet structure loaded with nanoparticles and displays two phases, typical spinel oxide and alloy. Scale bars: (A) 500 nm, (B) 200 nm and (inset) 5 1/nm.

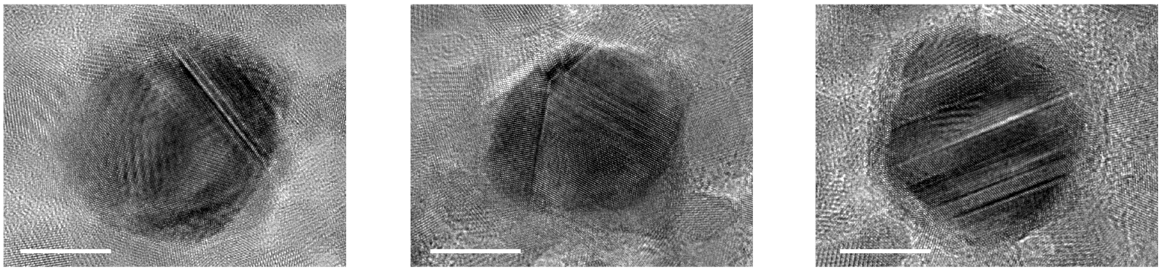

Fig. S26. The high-resolution TEM images of anchored CrCo nanoparticles at various regions. Twin structures are distinctly observed within these nanoparticles, attributing to the existence of uneven stress fields in anchored CrCo nanoparticles. Scale bar: 10 nm.

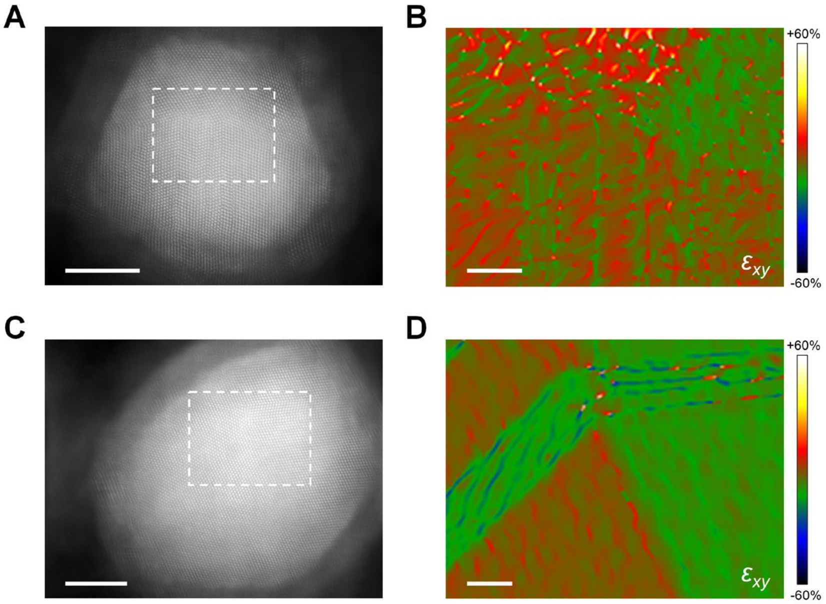

Fig. S27. The atomic HAADF-STEM images for anchored CrCo nanoparticles at various regions and corresponding analysis of strain fields ε_{xy} via GPA method. The selected areas of strain analysis are marked as white dashed boxes. These anchored CrCo nanoalloys exhibit twin crystal structures and inhomogeneous stress distributions within nanoalloys. Scale bars: (A, C) 5 nm, (B, D) 2 nm.

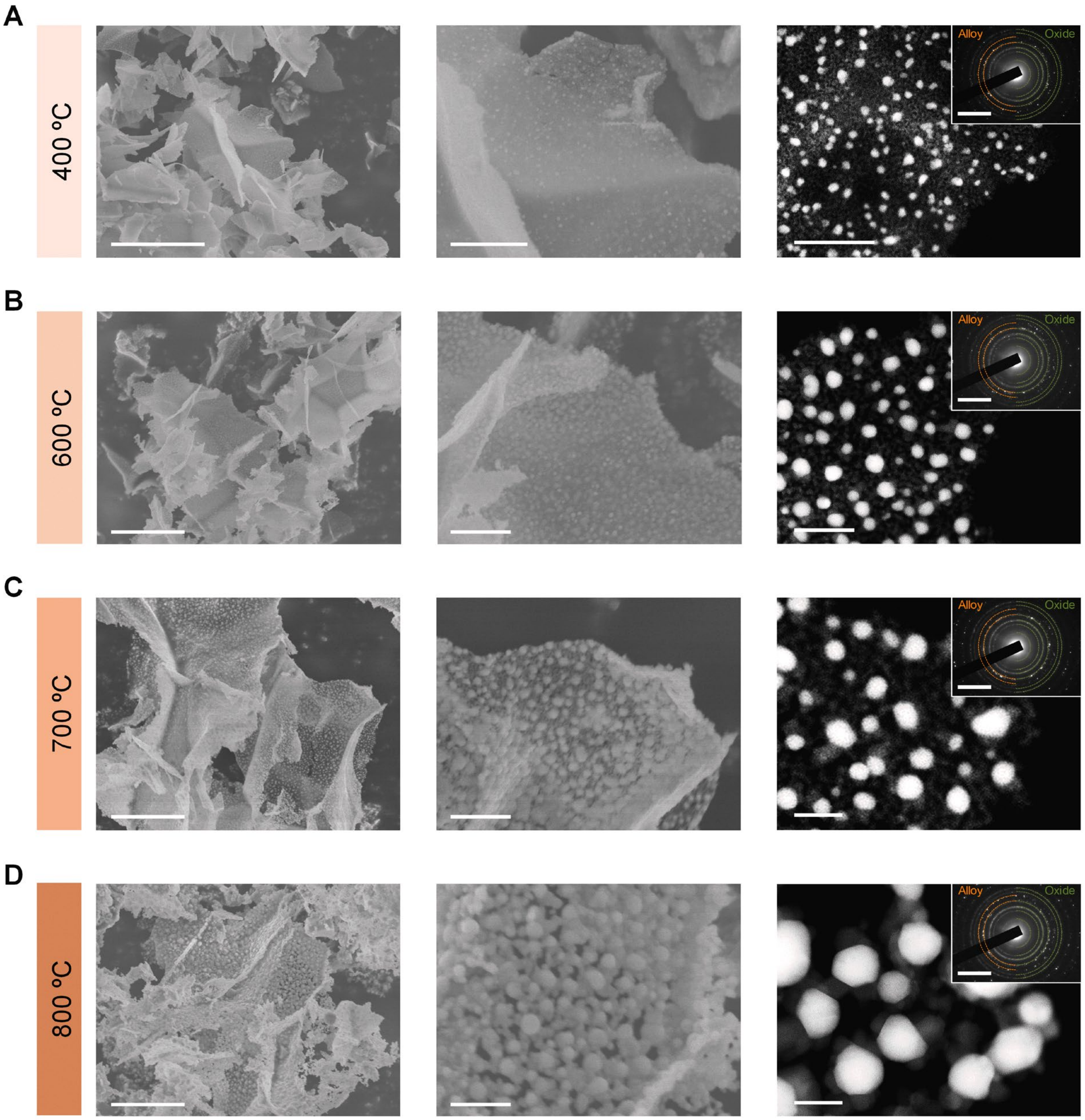

Fig. S28. The morphology characterization of size regulation for precipitated HEA nanoparticles via controlling reduction temperature. (A) 400 °C, (B) 600 °C, (C) 700 °C, (D) 800 °C. All samples exhibit the nanosheet structure loaded with nanoparticles. With the increasing of reducing temperature, a gradual increase in the size of precipitated nanoparticles is detected. At high temperatures, a slight collapse of lamellar substrate happens, resulting from the increase of precipitated phase. Scale bars: (Left) 2 μm, (Middle) 200 nm, (Right) 100 nm and (inset) 5 1/nm.

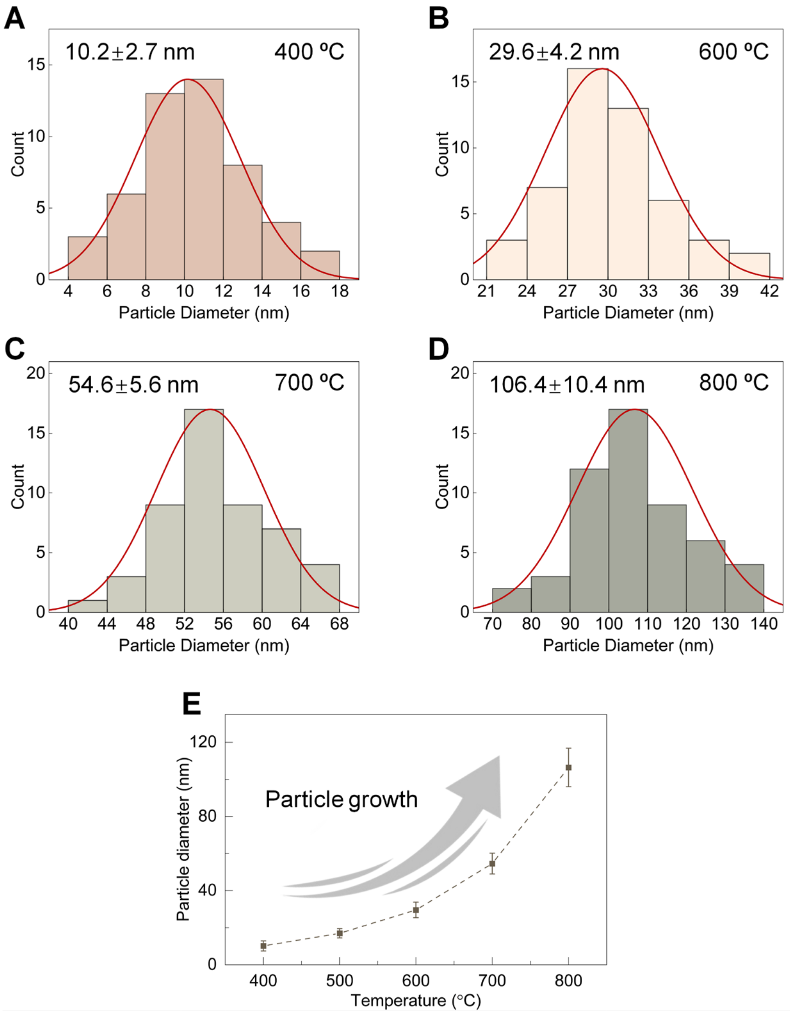

Fig. S29. The size distribution statistics for precipitated HEA nanoparticles at various reduction temperatures. As the target temperature increases from 400 ºC to 800 ºC, the precipitated nanoparticle gradually grows from the nanoscale to the submicron scale, demonstrating great size tunability for the prepared high-entropy system.

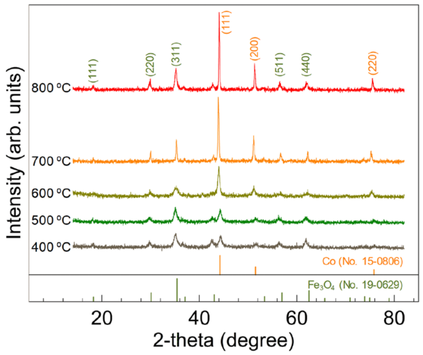

Fig. S30. The XRD patterns for precipitated HEA nanoparticles at various reduction temperatures. All samples exhibit both the spinel oxide and alloy phases, without any characteristic peaks for impurity. The diffraction peaks for alloy become sharp and narrow with increasing temperature, indicating the increase of both precipitated phase and nanoparticle diameter.

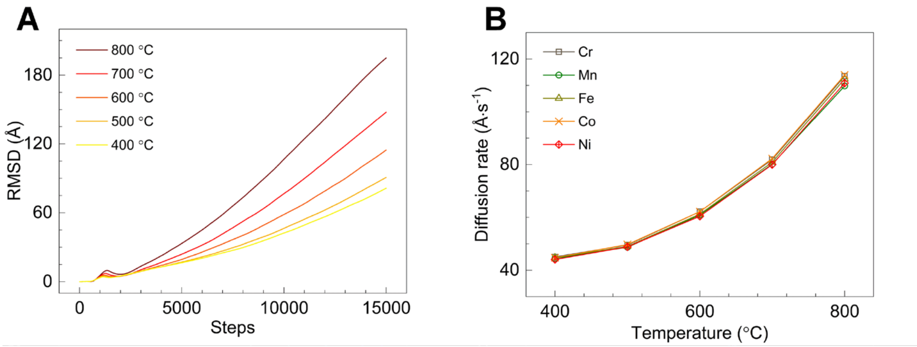

Fig. S31. The molecular dynamic simulation for the precipitation process of anchored HEA nanoparticles at different reduction temperatures. (A) the mean curves of root-mean-square displacement (RMSD) of five elements under various temperature conditions for 15000 steps, (B) diffusion rates of Cr, Mn, Fe, Co and Ni atoms at different temperatures. The RMSD curves describe the reason for the increase in diameter of HEA nanoparticles with rising temperature. The differences of diffusion rates for five elements are slight at each reducing temperature, avoiding the phase separation.

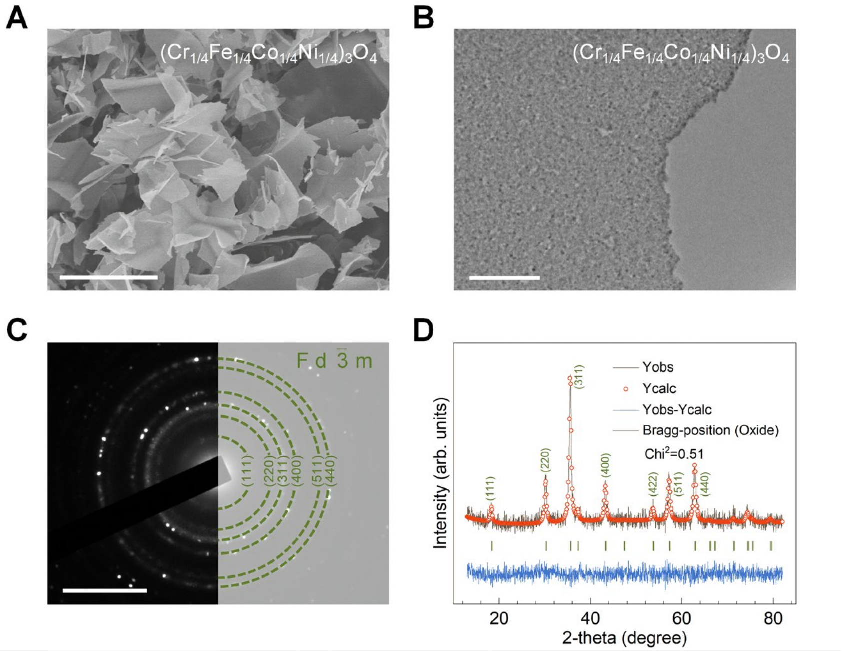

Fig. S32. The morphology and crystal characterization of $(Cr_{1/4}Fe_{1/4}Co_{1/4}Ni_{1/4})_3O_4$ nanosheets. This sample exhibits the thin nanosheet structure and typical spinel oxide phase. Scale bars: (A) 5 μm, (B) 200 nm, (C) 5 1/nm.

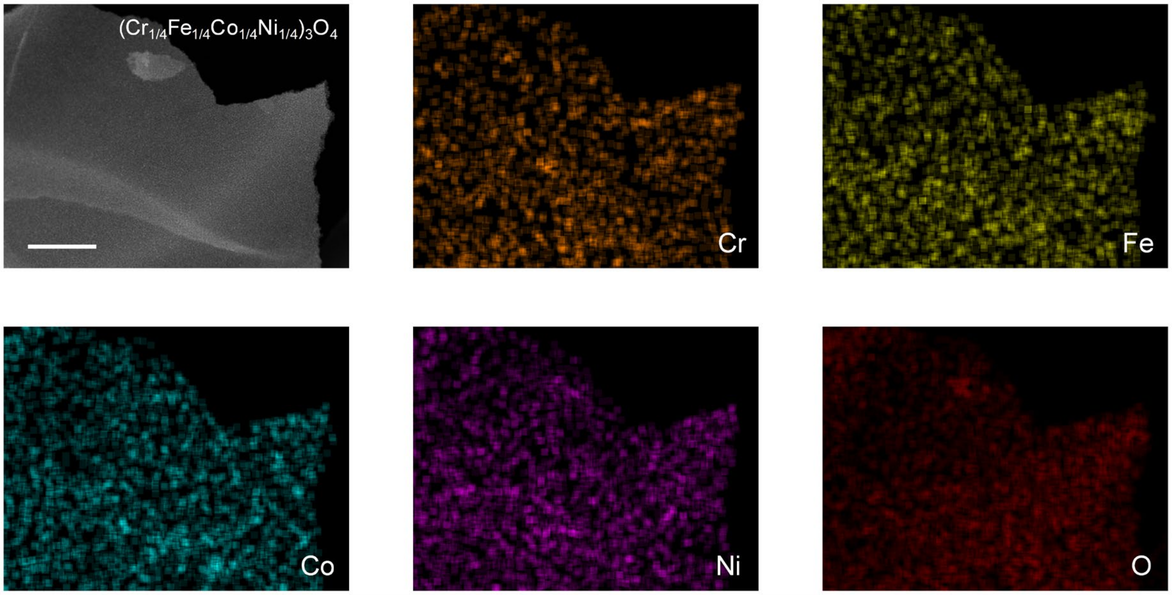

Fig. S33. The energy-dispersive X-ray spectroscopy (EDS) mapping of $(Cr_{1/4}Fe_{1/4}Co_{1/4}Ni_{1/4})_3O_4$ nanosheets. The elements of Cr, Fe, Co, Ni, and O are uniformly distributed within nanosheets, without any elemental separation. Scale bar: 500 nm.

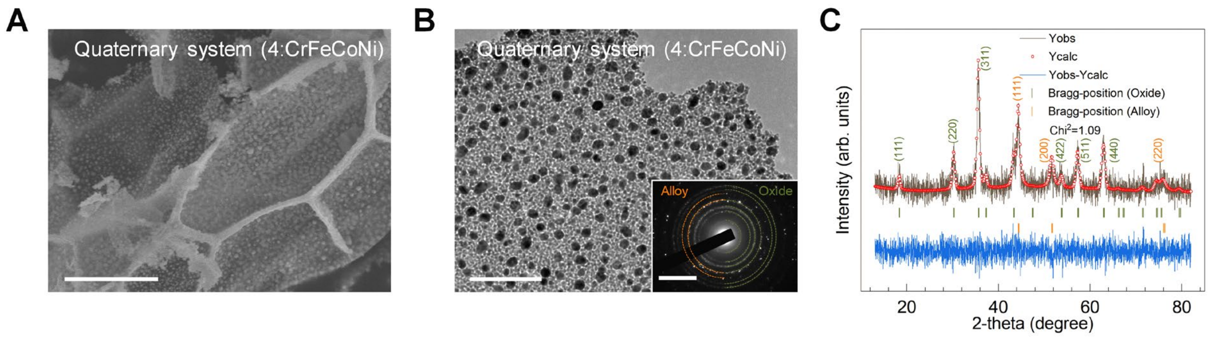

Fig. S34. The morphology and crystal characterization of the quaternary system (CrFeCoNi). This system features a thin nanosheet structure embedded with nanoparticles and displays two phases without any impurity. Scale bars: (A) 500 nm, (B) 200 nm and (inset) 5 1/nm.

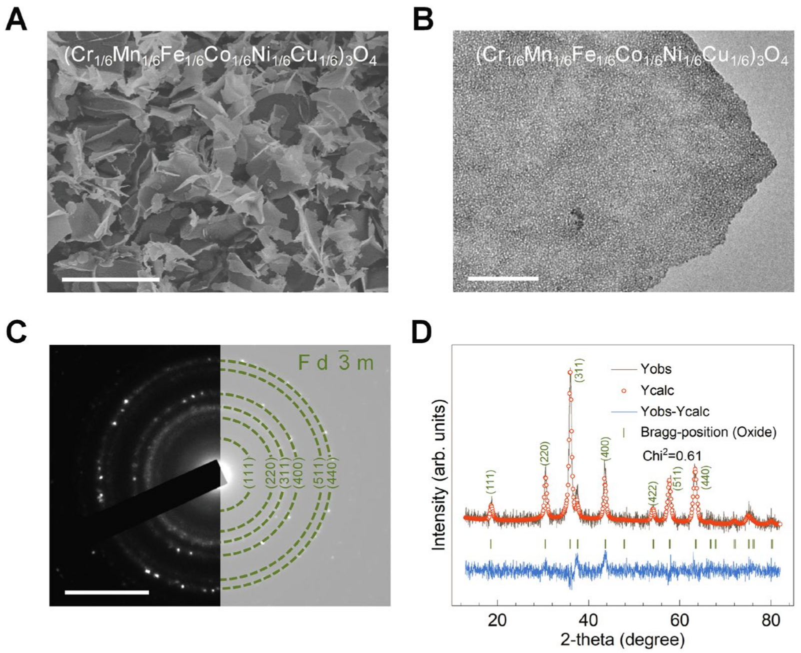

Fig. S35. The morphology and crystal characterization of $(Cr_{1/6}Mn_{1/6}Fe_{1/6}Co_{1/6}Ni_{1/6}Cu_{1/6})_3O_4$ nanosheets. This sample exhibits the thin nanosheet structure and typical spinel oxide phase. Scale bars: (A) 5 μm, (B) 200 nm, (C) 5 1/nm.

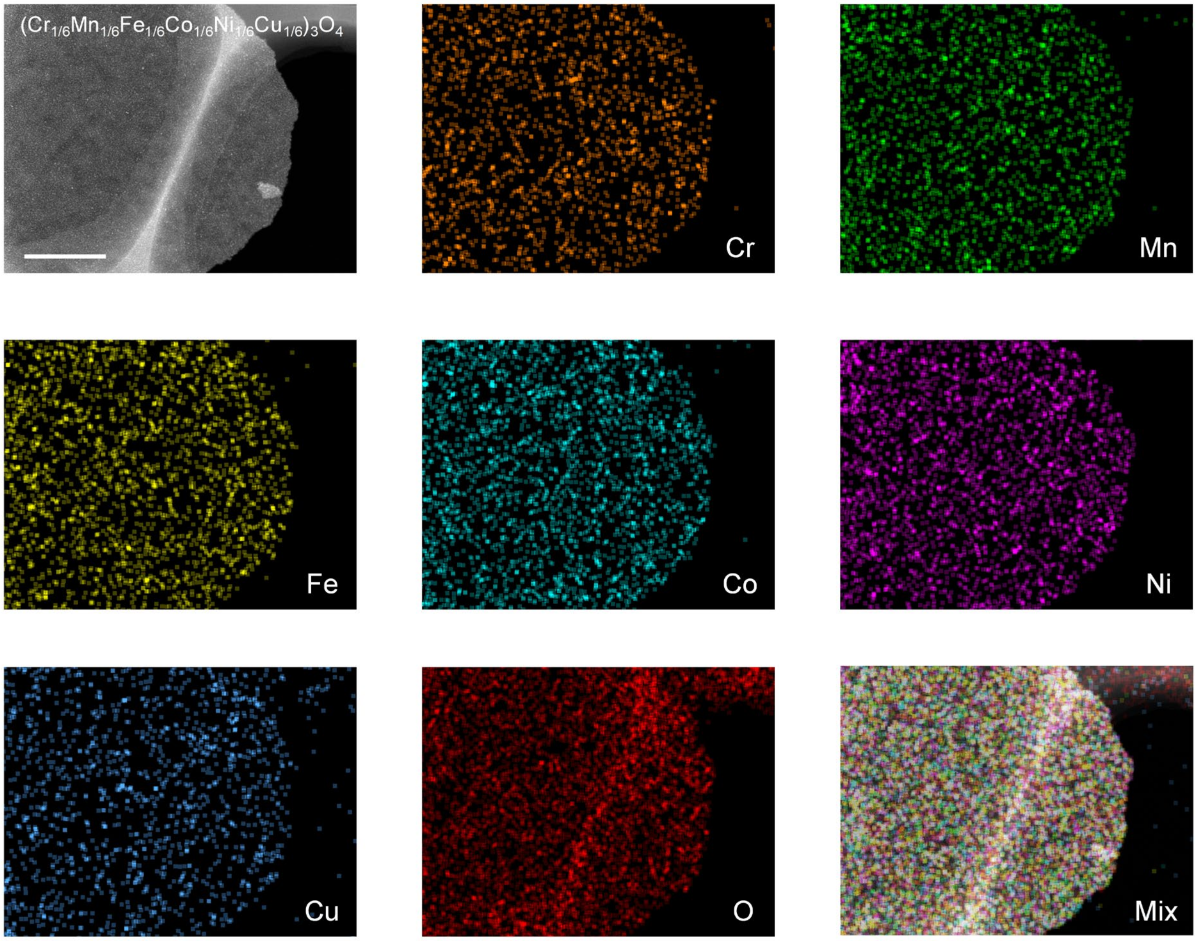

Fig. S36. The energy-dispersive X-ray spectroscopy (EDS) mapping of $(Cr_{1/6}Mn_{1/6}Fe_{1/6}Co_{1/6}Ni_{1/6}Cu_{1/6})_3O_4$ nanosheets. The elements of Cr, Mn, Fe, Co, Ni, Cu, and O are uniformly distributed within nanosheets, without any elemental separation. Scale bar: 500 nm.

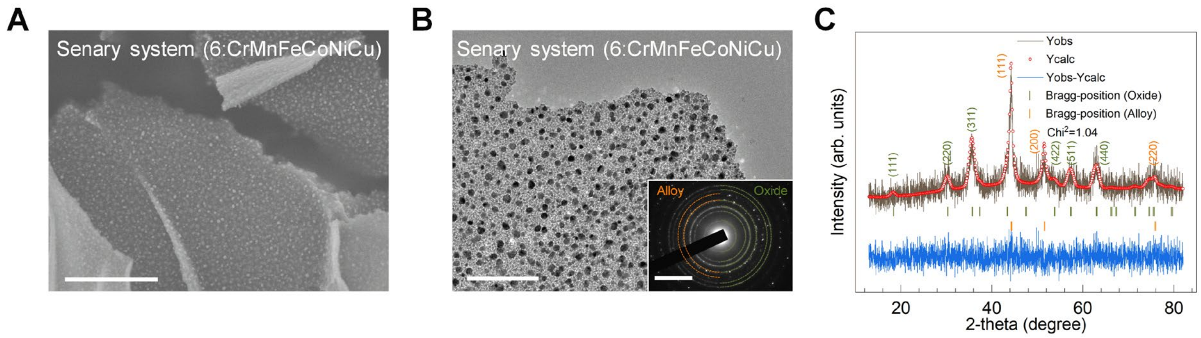

Fig. S37. The morphology and crystal characterization of the senary system (CrMnFeCoNiCu). The two-dimensional nanosheet structure incorporating nanoparticles is detected, comprising two phases, the conventional spinel oxide and alloy. Scale bars: (A) 500 nm, (B) 200 nm and (inset) 5 1/nm.

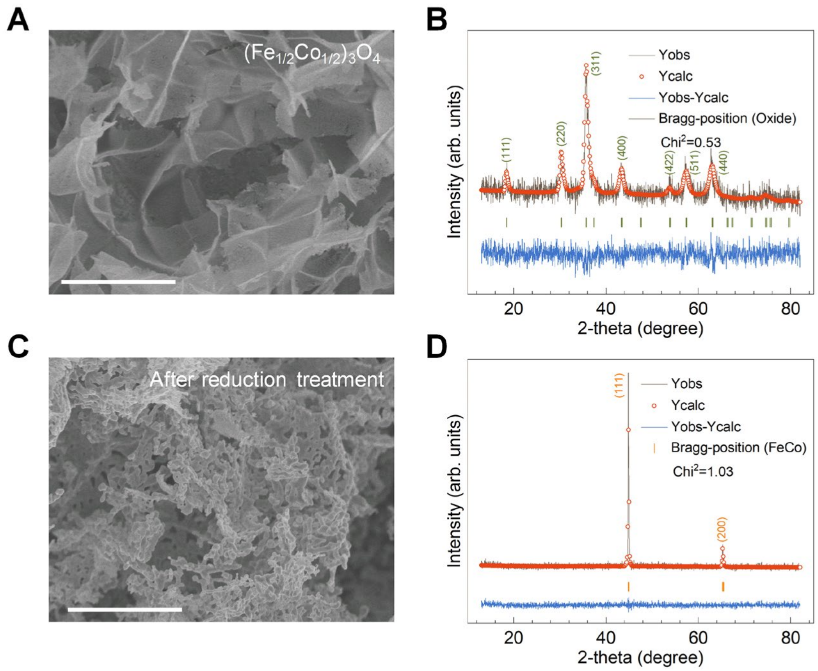

Fig. S38. The morphology and crystal characterization of $(Fe_{1/2}Co_{1/2})_3O_4$ nanosheets before and after the thermal reduction treatment. Before the reducing treatment, the sample exhibits a thin nanosheet shape with typical spinel oxide phase. While after the treatment, the nanosheet structure collapses, and only the alloy phase is observed in corresponding XRD pattern, demonstrating that the oxide precursors are completely converted to alloys. Scale bar: 5 µm.

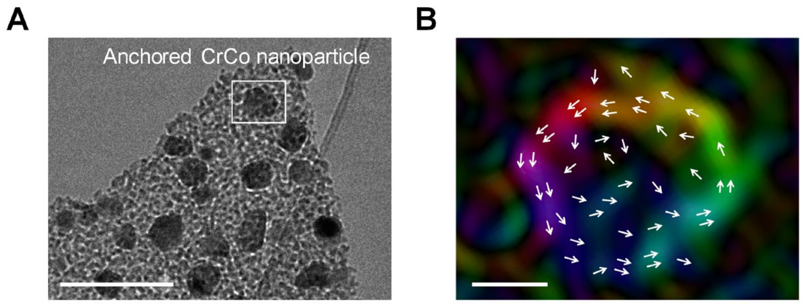

Fig. S39. Off-axis electron holography images of internal magnetization distribution for a single CrCo nanoalloy within the binary system. It exhibits a disordered distribution of magnetic moments. Scale bars: (A) 100 nm, (B) 10 nm.

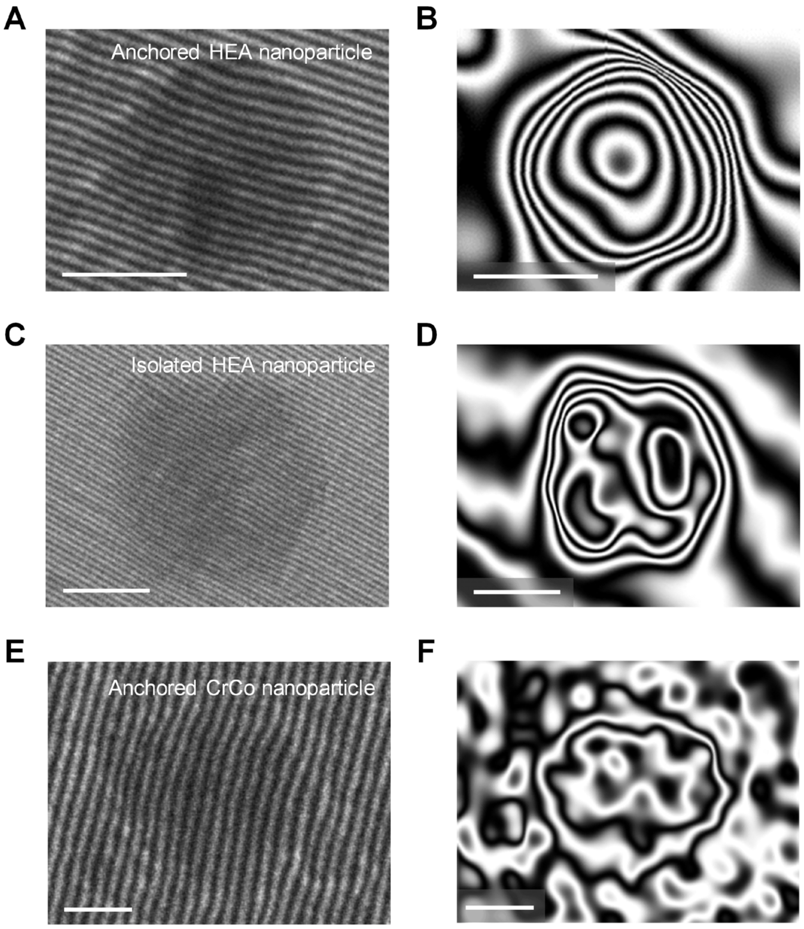

Fig. S40. Off-axis electron holography images of outer magnetic lines flux for single nanoalloys. (A, B) Anchored HEA nanoparticle, (C, D) isolated HEA nanoparticle, (E, F) anchored CrCo nanoparticle within binary system. For the anchored HEA nanoparticle, the majority of strong magnetic flux lines are confined within the nanoparticle, while for the isolated HEA nanoparticle or the anchored CrCo nanoparticle, the magnetic flux lines are either greatly leaked or twisted. Scale bar: 10 nm.

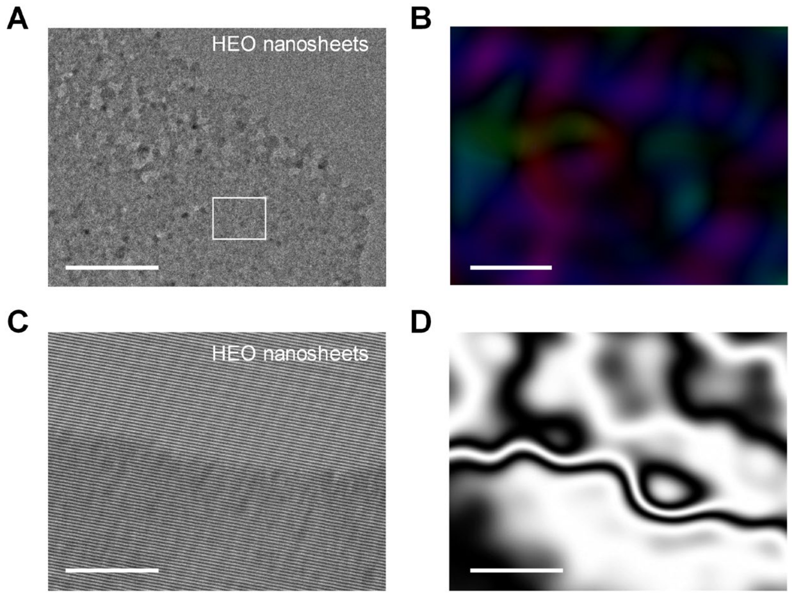

Fig. S41. Off-axis electron holography images of internal magnetization distribution and outer magnetic line flux for HEO substrates. (A, B) Internal magnetization configuration, (C, D) outer magnetic line flux. The HEO substrates exhibit the weak and disordered internal magnetic configuration, coupled with feeble magnetic stray field, indicating the negligible intrinsic magnetism. Scale bars: (A) 10 nm, (B) 10 nm, (C) 100 nm, (D) 100 nm.

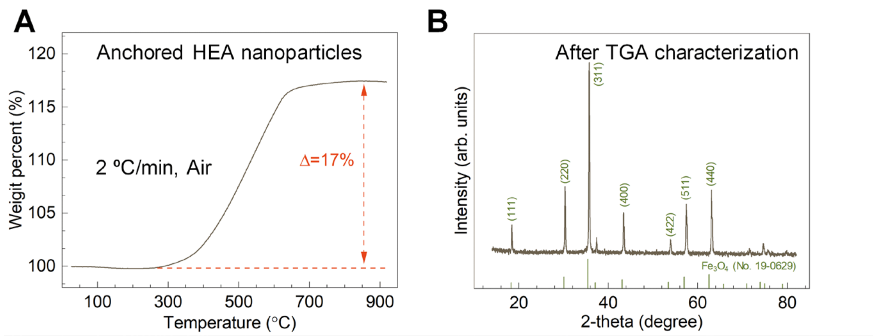

Fig. S42. The thermogravimetric (TG) analysis of anchored HEA nanoparticles. (A) The curve of mass variation from room temperature to 900 ºC with a heating ratio of 2 ºC/min in air, (B) The XRD pattern after TGA characterization. The difference of mass weight is about 17% when HEO-supported HEA nanosheets are fully oxidated.

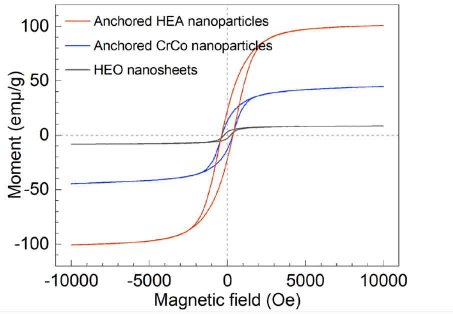

Fig. S43. Magnetization (M–H) curves for anchored HEA nanoparticles, anchored CrCo nanoparticles, and HEO nanosheets. In comparison to anchored HEA nanoparticles, the anchored CrCo nanoparticles and HEO nanosheets exhibit weak magnetism with low saturation magnetizations.

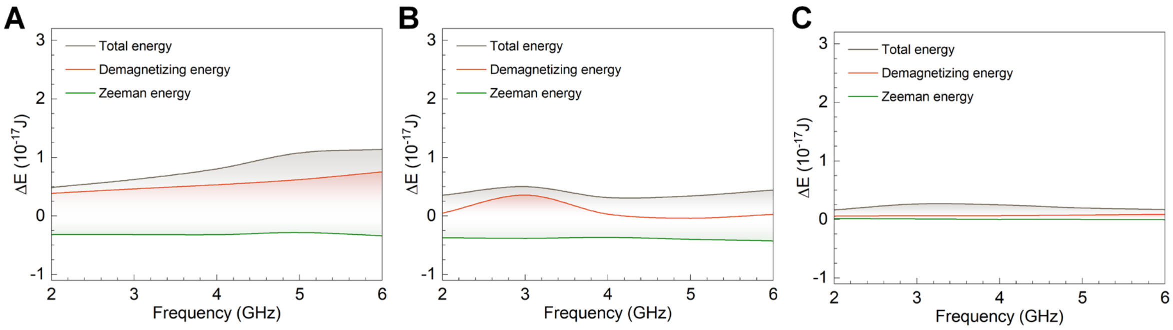

Fig. S44. Total energy, demagnetizing energy and Zeman energy difference for (A) isolated HEA nanoparticles, (B) anchored CrCo nanoparticles, (C) HEO nanosheets within 2-6 GHz through micromagnetic simulation. In comparison to anchored HEA nanoparticles, all the samples shown here exhibit weak responses to high-frequency magnetic field.

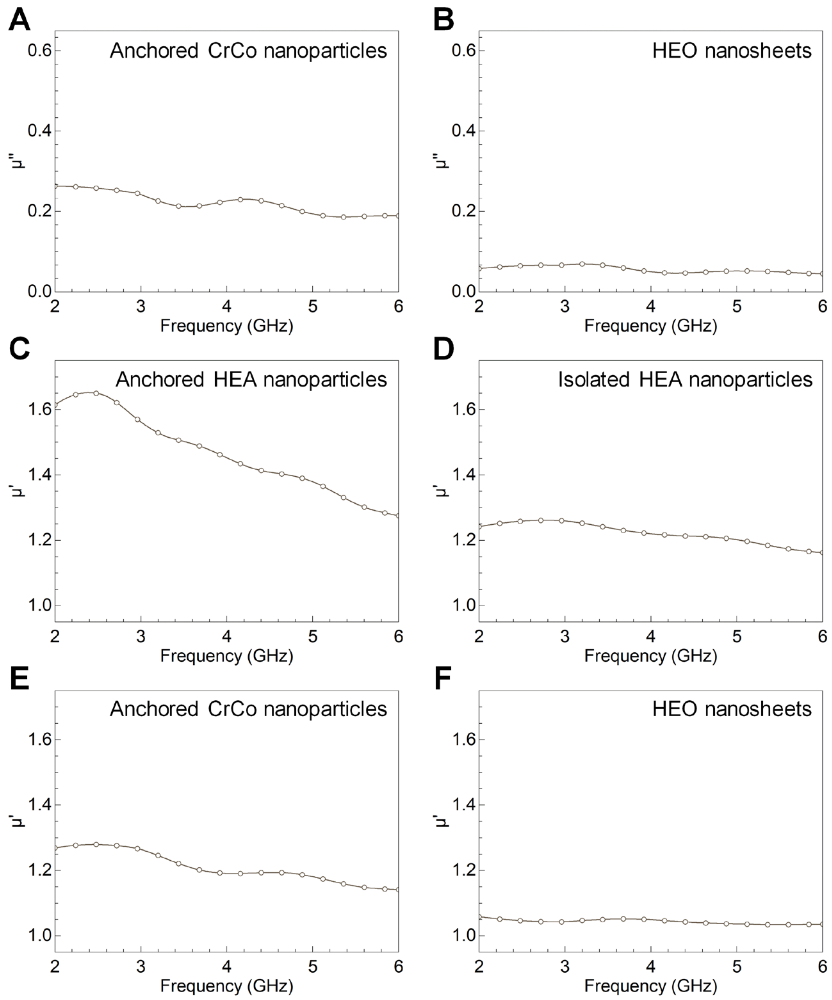

Fig. S45. The magnetic permeability parameters within the frequency range of 2-6 GHz. μ'' values for (A) anchored CrCo nanoparticles, (B) HEO nanosheets, and μ' values for (C) anchored HEA nanoparticles, (D) isolated HEA nanoparticles, (E) anchored CrCo nanoparticles, (F) HEO nanosheets. The anchored HEA nanoparticles exhibit notable enhancement in magnetic permeabilities (μ' and μ'') in comparison to isolated HEA and anchored CrCo nanoparticles. In addition, the negligible values of magnetic permeabilities are detected in HEO nanosheets, indicating weak magnetism.

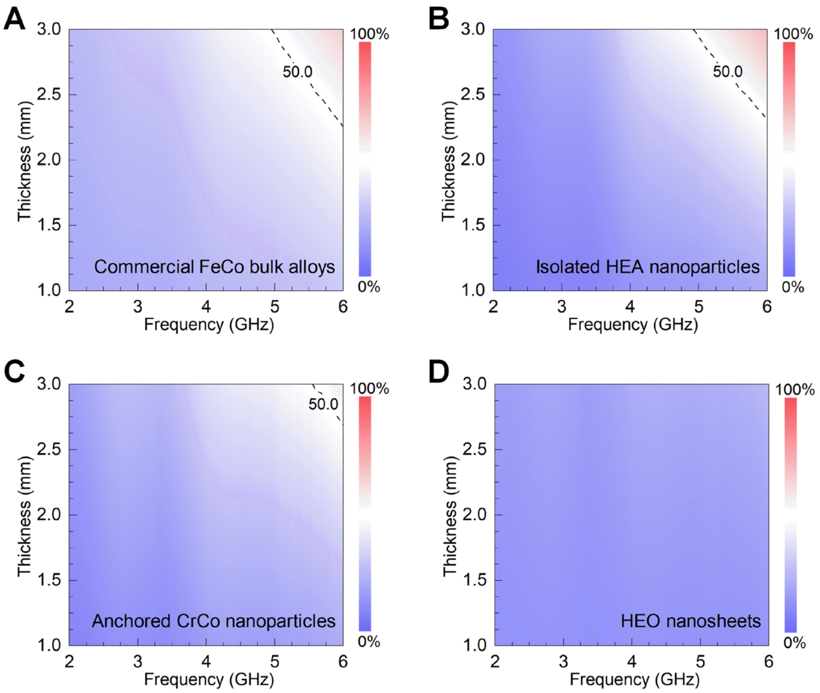

Fig. S46. 2D microwave absorption intensity mapping under room temperature within the frequency range of 2-6 GHz and the thickness from 1 to 3 mm. (A) Commercial FeCo bulk alloys, (B) isolated HEA nanoparticles, (C) anchored CrCo nanoparticles, (D) HEO nanosheets. In comparison to the anchored HEA nanoparticles, all the samples exhibit insufficient electromagnetic wave absorption performance within the standard 5G spectrum.

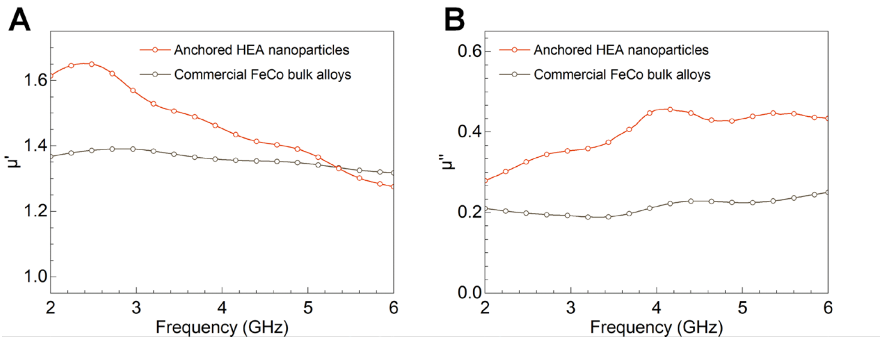

Fig. S47. The comparison of complex permeability between anchored HEA nanoparticles and commercial FeCo bulk alloys. (A) μ', (B) μ''. It exhibited that the prepared anchored HEA nanoparticles display remarkable enhancements in permeability values compared to commercial FeCo bulk alloys.

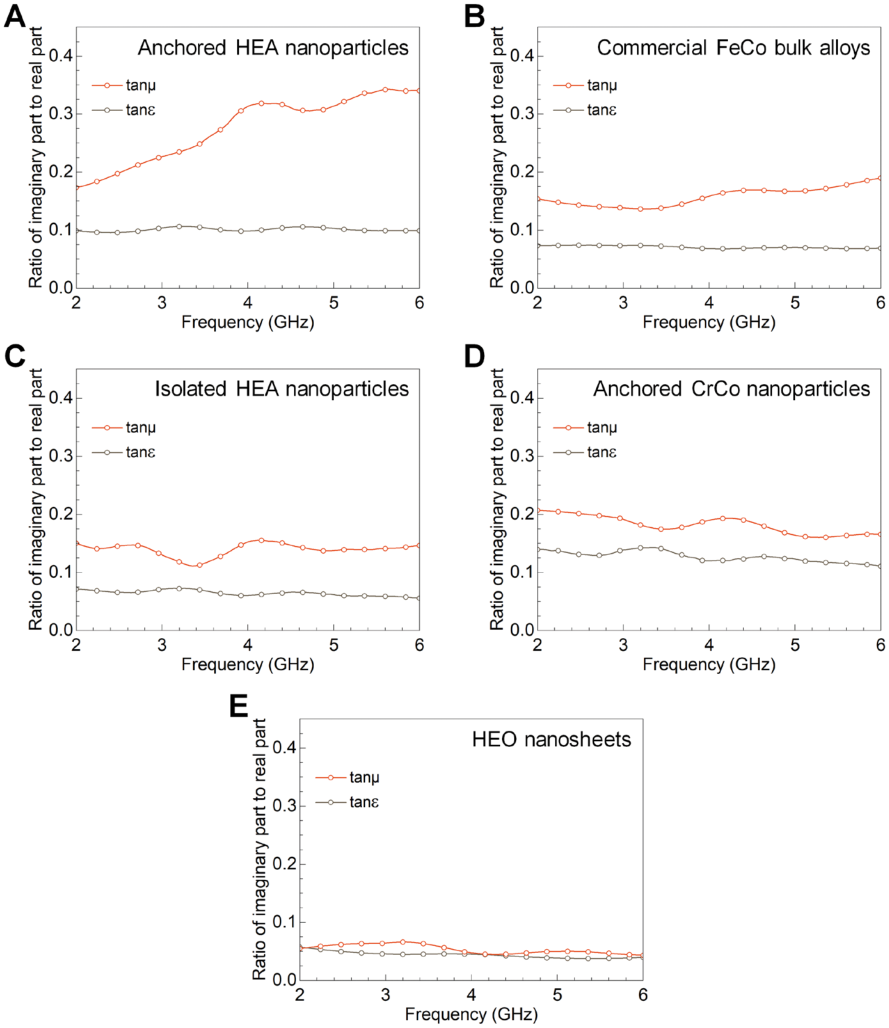

Fig. S48. The comparison of magnetic loss (*tanμ*) and dielectric loss (*tanε*). (A) anchored HEA nanoparticles, (B) commercial FeCo bulk alloys, (C) isolated HEA nanoparticles, (D) anchored CrCo nanoparticles, (E) HEO nanosheets. For these samples, excluding HEO nanosheets, magnetic loss exhibits the dominant position in electromagnetic dissipation process. HEO nanosheets show neither sufficient magnetic loss nor dielectric loss capabilities.

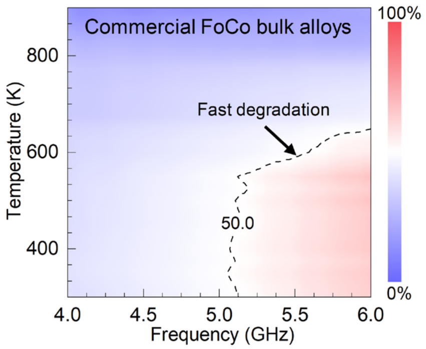

Fig. S49. 2D absorption intensity mapping for commercial FeCo bulk alloys with temperature from 300 to 900 K in frequency range of 2-6 GHz (thickness: 2 mm). In comparison to anchored HEA nanoparticles, the fast degradation of attenuation capability is advanced to about 600 K for commercial FeCo bulk alloys.

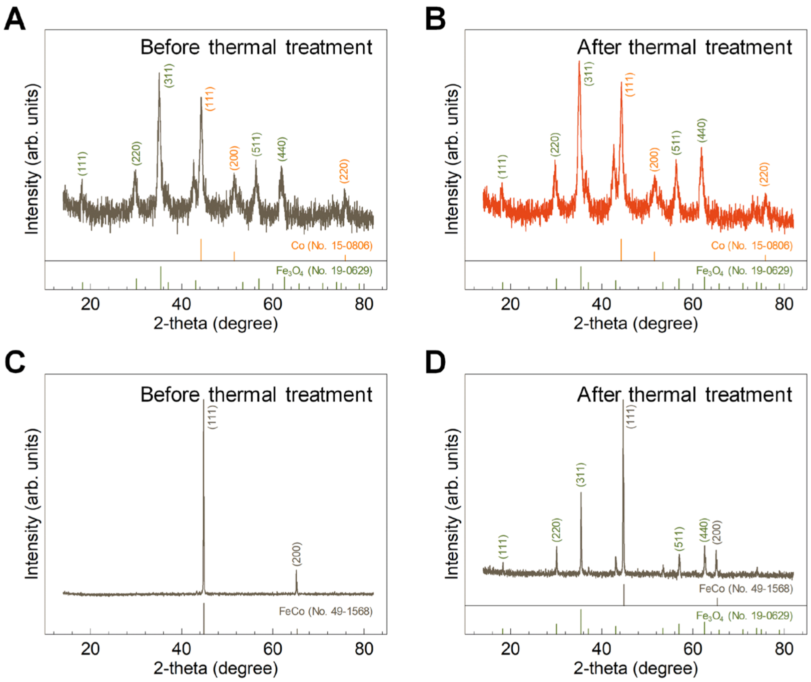

Fig. S50. The XRD patterns before and after the thermal treatment in air at 600 K for 20 h. (A, B) anchored HEA nanoparticles, (C, D) commercial FeCo bulk alloys. The XRD pattern is almost unchanged for anchored HEA nanoparticles after the thermal treatment, while an obvious oxidation phenomenon is detected for commercial FeCo bulk alloys after heating in air for 20 h.

Supplementary Tables

Supplementary Table 1. The EDS element content for the high-entropy system that nano HEAs anchored on HEO nanosheets.

Cr at.%	Mn at.%	Fe at.%	Co at.%	Ni at.%
**Total system**				
20.4	19.2	19.3	20.5	20.6
19.1	19.3	19.4	21.5	20.7
19.6	18.3	20.2	20.4	21.5
**Precipitate**				
8.5	11.2	17.7	33.2	29.4
9.7	10.8	17.2	30.8	31.5
10	9.3	19.8	31.7	29.2
**Substrate**				
31.2	29.6	21.4	9.2	8.6
30.2	31.4	17.7	9.5	11.2
28.3	31.7	20.8	8.9	10.3

[a]: The selected areas of precipitate and substrate are marked in fig. S10.

Supplementary Table 2. Comparison of effective absorption bandwidth (EAB) within the frequency range of 2–6 GHz and corresponding thickness for the electromagnetic absorption properties of various absorbers.

Samples	Thickness mm	EAB GHz	Ref.
**High entropy oxide**			
$(MnNiCuZn)_{1-x}Co_xFe_2O_4$	3.7	1.8	39
$(Ca_{0.2}Sr_{0.2}Ba_{0.2}La_{0.2}Pd_{0.2})TiO_3$	4.0	1.2	40
$(Mg_{0.2}Mn_{0.2}Fe_{0.2}Co_{0.2}Ni_{0.2})Fe_2O_4$	3.7	1.0	41
$(Cr_{0.2}Mn_{0.2}Cu_{0.2}Fe_{0.2}Ni_{0.2})_3O_4$	4.2	1.0	42
$(Co_{0.2}Mn_{0.2}Ni_{0.1}Cu_{0.4}Zn_{0.1})Fe_2O_4$	4.5	0.2	43
$(Fe_{1/3}Co_{1/6}Ni_{1/6}Cr_{1/6}Mn_{1/6})_3O_4$	4.2	0.1	44
$(Mn_{0.2}Co_{0.2}Ni_{0.2}Fe_{0.2}Ce_{0.2})_3O_4$	4.2	0.5	45
$(CoCrFeMnNi)_3O_4$	4.0	0.7	46
$(Fe_{0.2}Co_{0.2}Ni_{0.2}Cr_{0.2}Mn_{0.2})_3O_4$	4.3	1.2	47
**High entropy alloys**			
$FeCoNiAlCr_{0.9}$	3.0	1.2	48
FeCoNiTiCr	2.3	1.0	49
FeCoNiCrAl	2.7	1.4	50
FeCoNiCuTi	2.9	0.9	51
$FeCoNiMn_{0.5}Al_{0.2}$	3.0	1.8	52
FeCoNiCuZn	2.5	1.4	53
FeCoNiCuZn	2.5	1.1	54
$FeCoNiMnSi_x$	2.8	1.7	55
$FeCoNiCrAl_{0.8}$	2.7	0.8	56
**Magnetic composites**			
FeCo/FeCoNi	3.5	1.7	57
$Co_{0\cdot 8}Ni_{0.2}$	3.7	1.4	58
FeCo	3.2	1.3	59
FeCoNiAl	3.3	2.1	60
NiFe	3.0	1.4	61
NiFe@NFCs	3.0	1.9	62
$NiCo@C_3N_4$	3.7	1.9	63
$Sm_2Co_{17}/Co(Sm)$	3.3	1.6	64
Ni@C-CoNi	3.6	2.2	65
**Carbon-based composites**			
NiS/CNFs/porous carbon	4.5	1.5	66
$NiCo_2S_4/C$	4.0	1.9	67
Co SAs/porous carbon	4.5	2.0	68
NiAl-LDH/Graphene	3.6	1.2	69

Co@CNTs/PC	4.3	1.4	70
CNT aerogels	4.2	1.8	71
MXene microsphere	3.8	1.4	72
MXene/PI aerogel	3.5	2.0	73
HPCNs	4.0	1.5	74
**This work**			
anchored HEA nanoparticles	2.0	2.7	-

[b]: The reference number links to the main reference list.
